# Comparative effectiveness and acceptability of internet-based psychological interventions on depression in young people: a systematic review and network meta-analysis

**DOI:** 10.1186/s12888-025-06757-9

**Published:** 2025-04-02

**Authors:** Baijun Chen, Jialong Li, Yuxin Qi, Honghui Mao, Yihui Liu, Wenting Wang

**Affiliations:** 1 Department of Neurobiology, Basic Medical Science Academy, Air Force Medical University, Xi’an, 710032 China; 2 Key Lab of Modern Teaching Technology, Ministry of Education, Shaanxi Normal University, Xi’an, 710062 China

**Keywords:** Depression, Internet-based psychological interventions, Comparative effectiveness, Acceptability, Network meta-analysis

## Abstract

**Background:**

Depression represents a major global public health challenge, particularly among young individuals aged between 10 and 25. This age bracket is notably critical, as the onset of depression during these years tends to be more severe and consequential. In response to the growing demand for mental health services, internet-based psychological interventions have gained traction as a flexible and convenient alternative to traditional face-to-face treatment. This systematic review and network meta-analysis aims to rigorously assess the comparative efficacy and acceptability of internet-based psychological interventions in addressing depression within the young population over the past three decades.

**Methods:**

We conducted a comprehensive search of seven electronic databases for eligible randomized controlled trials published from January 1995 to July 2024. The literature screening process adhered to the principles of population, intervention, comparator, outcome, and study design. The quality of the included studies was assessed using the Cochrane Risk of Bias Assessment Tool. To evaluate the ranking probability of each intervention, we calculated the surface under the cumulative ranking curve values. Network meta-analysis (NMA) was conducted using RStudio and Stata software.

**Results:**

The NMA incorporated a total of 27 studies involving 3,451 participants. Among these studies, 18 assessed internet-based cognitive behavioral therapy (iCBT) interventions, whereas 12 employed a waitlist as a control group. At the end of the interventions, internet-based acceptance and commitment therapy (iACT), iCBT, internet-based dialectical behavior therapy (iDBT), and internet-based psychodynamic therapy (iPDT) all demonstrated statistically significant reductions in depression scores. Notably, no intervention measure was found to be statistically more acceptable than the others.

**Conclusions:**

Our NMA indicated that iDBT appeared to be more effective, whereas internet-based mindfulness-based therapy (iMBT) may be more acceptable. These findings offered preliminary evidence regarding the comparative effectiveness and acceptability of internet-based psychological interventions in treating depression among young people. However, the limited number of eligible studies underscored the importance and necessity of further research to evaluate novel intervention measures.

**Trial registration:**

The study was registered with the International Prospective Register of Systematic Reviews (PROSPERO), with the registration number CRD42024580958.

**Supplementary Information:**

The online version contains supplementary material available at 10.1186/s12888-025-06757-9.

## Introduction

Depression is a multifaceted mental health disorder characterized by persistent disinterest in daily activities and accompanied by a range of emotional, cognitive, physical, and behavioral symptoms [1]. It is recognized as one of the leading causes of global disability, and in severe cases, it can lead to suicidal thoughts or behaviors [2]. Depression significantly impairs the quality of life for patients and their families and imposes substantial economic burdens on society [3].

Individuals aged 10–25 are at a pivotal stage of development and represent a population with a high prevalence of depression [2, 4]. Compared to late-onset depression, early-onset depression in this population often leads to more severe outcomes, including substance abuse, physical illness, social dysfunction, and even self-harm or suicidal behavior [5, 6]. Additionally, young people with depression have a higher propensity for relapse [5]. Therefore, there is an urgent need for effective intervention strategies to treat depression in this population.

Concerns regarding the safety of antidepressants have led clinical guidelines to recommend psychological interventions as the first-line treatment for young patients [6, 7]. Researches have shown that psychotherapies, including cognitive behavioral therapy (CBT), behavioral activation (BA), mindfulness-based therapy (MBT), and acceptance and commitment therapy (ACT), have demonstrated positive effects in treating depression [8, 9]. However, a meta-analysis revealed that only 36% of adolescents received treatment for depression [10]. Cognitive limitations, time constraints, social stigma, and deficiencies in mental health service infrastructure collectively impede many young individuals from availing face-to-face treatment opportunities [4, 10, 11]. Consequently, despite the recognized limitations of internet-based psychological interventions, such as security issues and difficulties in obtaining additional information [12], these digital platforms offer unparalleled advantages in terms of privacy, anonymity, and flexibility regarding time and location. These attributes making them a promising avenue deserving of further assessment and dissemination to enhance mental health support for adolescents [4].

Numerous investigations have been dedicated to evaluating the efficacy of internet-based cognitive behavioral therapy (iCBT) in addressing depression among youth. A review conducted by Wu et al. indicated a notable reduction in depression scores among adolescents following iCBT intervention [13]. Another meta-analysis revealed that, compared to the waiting list (WL) group, iCBT exhibited a moderate effect on depressive symptoms, with an effect size of g = 0.56 (95% CrI = 0.31–0.82, *p* < 0.001) [14]. Additionally, several studies have highlighted the superior efficacy of internet-based interventions over traditional control groups [15, 16]. Notwithstanding the demonstrated effectiveness of online interventions, adherence to treatment remains a pivotal challenge for young individuals seeking help for depression. The number of CBT sessions attended has been shown to be crucial. Specifically, participants who completed 10 or more sessions were found to be 2.5 times more likely to achieve an adequate treatment response compared to those who attended fewer than 10 sessions [17]. Research by Cuijpers et al. [18] demonstrated that guided self-help CBT encountered greater acceptance hurdles compared to treatment as usual (TAU), while unguided self-help CBT was even less acceptable than the WL. Melville’s review [19] provided further insights, noting that dropout rates in internet-based treatments varied significantly, ranging from 2 to 83%, with an average dropout rate of 35% and a median of 24%. These high dropout rates pose a significant barrier to patients fully benefiting from the potential therapeutic effectiveness of these interventions.

To our knowledge, limited research has systematically compared the efficacy and dropout rates of different internet-based psychotherapies among young people. Therefore, this study aims to explore the effectiveness and acceptability of various internet-based psychological interventions for young individuals with depression across the past three decades.

## Methods

### Search strategy and selection criteria

We conducted a search for eligible trials in the following electronic databases: Web of Science, PubMed, Embase, ScienceDirect, ProQuest, Cochrane Library, China National Knowledge Infrastructure (CNKI), and Wanfang databases. Our search was confined to randomized controlled trials (RCTs) published from 1995 to December 1, 2024, and was limited to literature in Chinese and English. The detailed keyword search strategy was provided in Additional File 1. Furthermore, to ensure no relevant studies were overlooked, we also reviewed existing reviews on the topic.

Based on the population, intervention, comparator, outcome, and study design (PICOS) principle [20], we formulated the following inclusion criteria for our study:

#### Populations

Eligible participants were adolescents and young adults aged 10 to 25 years who exhibited current symptoms of depression and had met or exceeded the threshold for at least mild depression, as assessed through validated clinical diagnostic interviews or standardized self-report scales. For studies involving student populations with an average sample age of 25 years or younger, inclusion was extended to encompass some participants above the age of 25 [21]. Conversely, studies focusing on individuals with other psychotic disorders, bipolar disorder, or active suicidality were excluded from the analysis.

#### Interventions

Interventions considered for inclusion were grounded in established psychotherapeutic theories, including but not limited to CBT, BA, and MBT. These interventions were required to be delivered via digital platforms, encompassing the internet, computers, or smartphones. Specific forms may include text, video, e-books, websites, applications, games, or artificial intelligence (AI) chatbots, which can be used individually or in combination. Interventions that involved face-to-face sessions or consisted solely of a single session were excluded from our analysis.

#### Comparators

Suitable control groups included: WL; active control (AC), such as supportive contact, non-specific counselling, or psychoeducation; and treatment as usual (TAU) or intervention based on another psychotherapeutic theory. Studies that incorporated face-to-face psychotherapies, such as CBT, BA, or MBT, within the control group were explicitly excluded from the review.

#### Outcomes

Effectiveness was defined as the difference in post-treatment scores between the intervention group and the control group. Acceptability was defined by the proportion of participants who discontinued the entire treatment course for various reasons [18]. Consequently, included research was expected to furnish scoring data pertaining to depressive symptoms, assessed via validated depression rating scales, for both intervention and control groups after treatment. Additionally, studies were required to report the number of dropouts in each treatment group. In scenarios where studies failed to provide these crucial data points, we endeavored to contact the corresponding authors to procure the necessary information. Studies for which authors did not respond were consequently excluded from the analysis.

#### Study designs

Studies must be RCTs and published in either Chinese or English. All of the researchers were Chinese and had good English reading ability.

### Selection procedure and data extraction

A preliminary search yielded a total of 12,349 potentially relevant literature entries. Following the removal of duplicates using EndNote 21 software, two independent researchers (BC and JL) proceeded to screen the remaining 9,491 articles. In cases where disagreements arose during the selection process, a third researcher (YQ) was consulted to facilitate consensus.

Data extraction was independently conducted by BC and JL using a standardized data extraction table. This table included the following details: study characteristics (authors, publication year, country), participant characteristics (mean age, gender ratio, sample size in each group, baseline depression scores), intervention details (type of intervention, duration, primary outcome measures), and outcome measures (mean and standard deviation of depression scale scores post-treatment, umber of dropouts in each group). Any inconsistencies or uncertainties encountered during data extraction were reviewed and resolved by HM, who cross-checked the original articles and engaged in discussions with BC and JL to ensure the accuracy and consistency of the extracted data.

### Risk of bias assessment

In accordance with the Cochrane Handbook for Systematic Reviews of Interventions [22], the risk of bias was evaluated across seven domains: random sequence generation (selection bias), allocation concealment (selection bias), blinding of participants and personnel (performance bias), blinding of outcome assessment (detection bias), incomplete outcome data (attrition bias), selective reporting (reporting bias), and other potential sources of bias. Two independent researchers (BC and JL) conducted the risk of bias assessment using Review Manager 5.4 [23]. Any disagreements were resolved through group discussions with a third researcher (HM).

### Data analysis

This study conducted a contrast-based Bayesian network meta-analysis (NMA) [22] to evaluate the comparative effectiveness and acceptability of different internet-based psychotherapies. Recognizing the presence of clinical and methodological heterogeneity across the included studies, a random-effects model [24] was employed to account for this diversity. For continuous outcomes, such as depression symptom scores, which were assessed using different depression scales across studies, standardized mean differences (SMD) were calculated to provide a uniform measure of effect, accompanied by 95% credible interval (CrI) to represent the estimated uncertainty. SMD was calculated by dividing the difference in post-intervention scores between the intervention and control groups by the pooled standard deviation (SD), with adjustments for small sample sizes using Hedge’s g method [22]. A negative SMD indicated superior effectiveness in the intervention group compared to the control group. In cases where SD was not reported in a study, it was estimated based on the 95% CrI or standard error (SE) to ensure data integrity. For dichotomous outcomes, such as all-cause discontinuation at the end of the intervention, odds ratios (OR) with 95% CrI [22] were used. This study prioritized results from intention-to-treat (ITT) analysis [25] or modified ITT over those from complete-case analysis to ensure the most robust and unbiased estimates.

This study employed Markov Chain Monte Carlo (MCMC) methods [26] to obtain the pooled estimates from the NMA. Specifically, four Markov chains were initialized with arbitrarily chosen initial values, and the convergence of these chains was assessed using the Brooks-Gelman-Rubin statistics [27]. To evaluate global inconsistency, the deviance information criterion (DIC) [28] was compared between the unrelated mean effects (UME) model and the consistency model. Local inconsistencies were examined using the node-splitting method [29]. For ranking the interventions, the surface under the cumulative ranking curve (SUCRA) values [30] were calculated for each treatment. Higher SUCRA values indicated a greater likelihood of the intervention being ranked at the top. Consequently, the interventions were ordered based on their respective SUCRA values.

The data preprocessing, including the conversion of raw data into relative effect formats and the generation of network plots, was carried out using StataMP 18 [31]. The subsequent analyses were performed using the *gemtc* package [32] in R version 4.4.1 [33].

## Results

### Study selection and inclusion

**Fig. 1:**
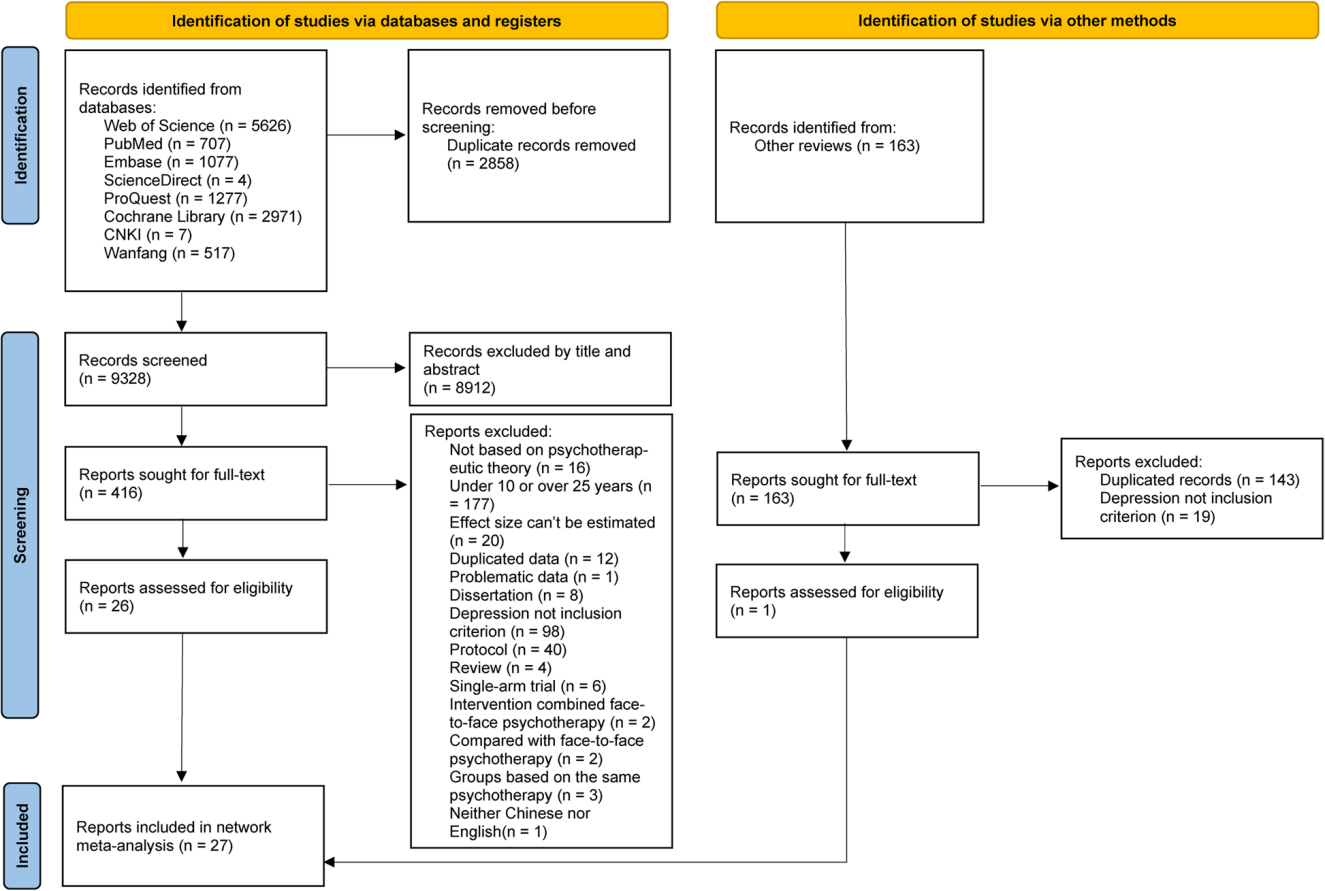
PRISMA flow diagram

A total of 12,186 records were initially identified through database searches. After removing duplicates, 9,328 records remained. Through the screening of titles and abstracts, 8,912 records were excluded, leaving 416 records for full-text evaluation. Upon thorough review of these full texts, 390 records were excluded based on predefined inclusion criteria, resulting in 26 records. Additionally, 163 records were identified from other meta-analyses, and after full-text assessment, only 1 record met the inclusion criteria. Finally, a total of 27 records [34–59] were included in the final analysis of this study. The detailed selection process is depicted in Fig. 1.

### Characteristics and risk of bias of included studies

This study included 27 studies involving a total of 3,345 participants. One of these studies [36] comprised three groups: guided internet-based behavioral activation (iBA), unguided iBA, and TAU. Another study [49] included four groups: the school-based CBT project OVK (Op Volle Kracht), the iCBT project SPARX (Smart, Positive, Active, Realistic, X-factor thoughts), a combination of OVK and SPARX, and a AC group. Since our NMA did not specifically focus on the effectiveness of guided versus unguided interventions or face-to-face interventions, we merged the data from the two iBA groups and excluded the two CBT groups containing OVK.

Therefore, all included studies were two-arm trials. A total of 8 distinct internet-based psychological interventions were identified, comprising iCBT, internet-based dialectical behavior therapy (iDBT), internet-based psychodynamic therapy (iPDT), internet-based mindfulness-based therapy (iMBT), internet-based acceptance and commitment therapy (iACT), internet-based solution-focused brief therapy (iSFBT), internet-based social cognitive theory (iSCT), and iBA. Of the 27 studies, 18 (66.67%) examined iCBT, while 12 (46.15%) used a WL as the control group. In total, 2,026 participants were assigned to the internet-based psychological intervention groups, and 1,425 participants were assigned to the control groups, which included treatment as TAU, WL, and AC. The specific characteristics of each study are detailed in Additional File 2, while the comprehensive description of the interventions is provided in Additional File 3.

The individual risk of bias and the overall risk of bias are shown in Additional File 4. Given the inherent characteristics of the interventions under study, the majority of the studies were found to have a high risk of performance bias.

### NMA of effectiveness

**Fig. 2:**
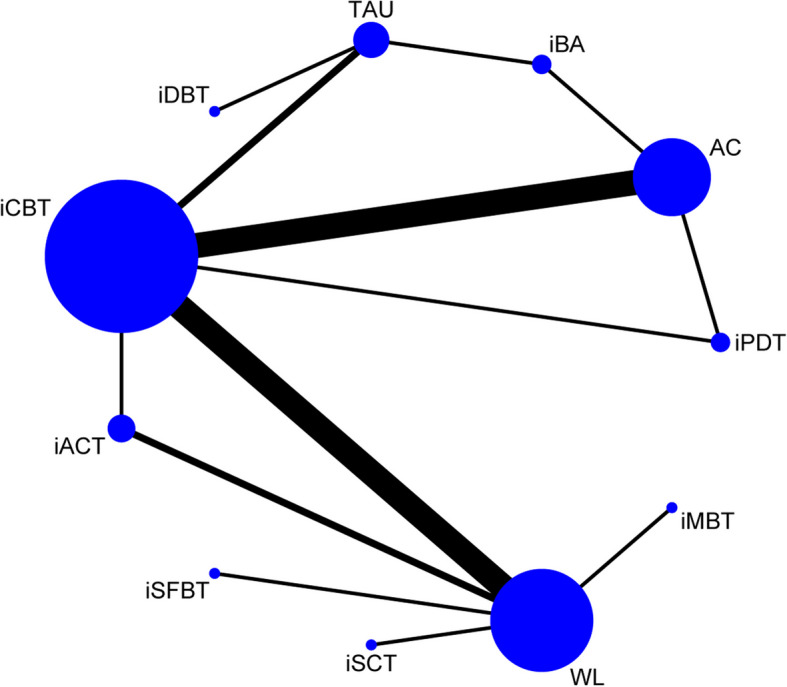
Network plot of effectiveness

The network plot of effectiveness is displayed in Fig. 2, where the size of the nodes and the thickness of the edges are weighted according to the number of participants and the frequency of comparisons, respectively. The Brooks-Gelman-Rubin diagnosis plot (Additional File 5) indicates that our consistency model achieved good convergence. We did not identify any significant global inconsistency, as the DIC was similar to the DIC in the UME model (57.87 vs. 56.66). The node-splitting method also did not detect any significant local inconsistencies (Additional File 6).

**Fig. 3:**
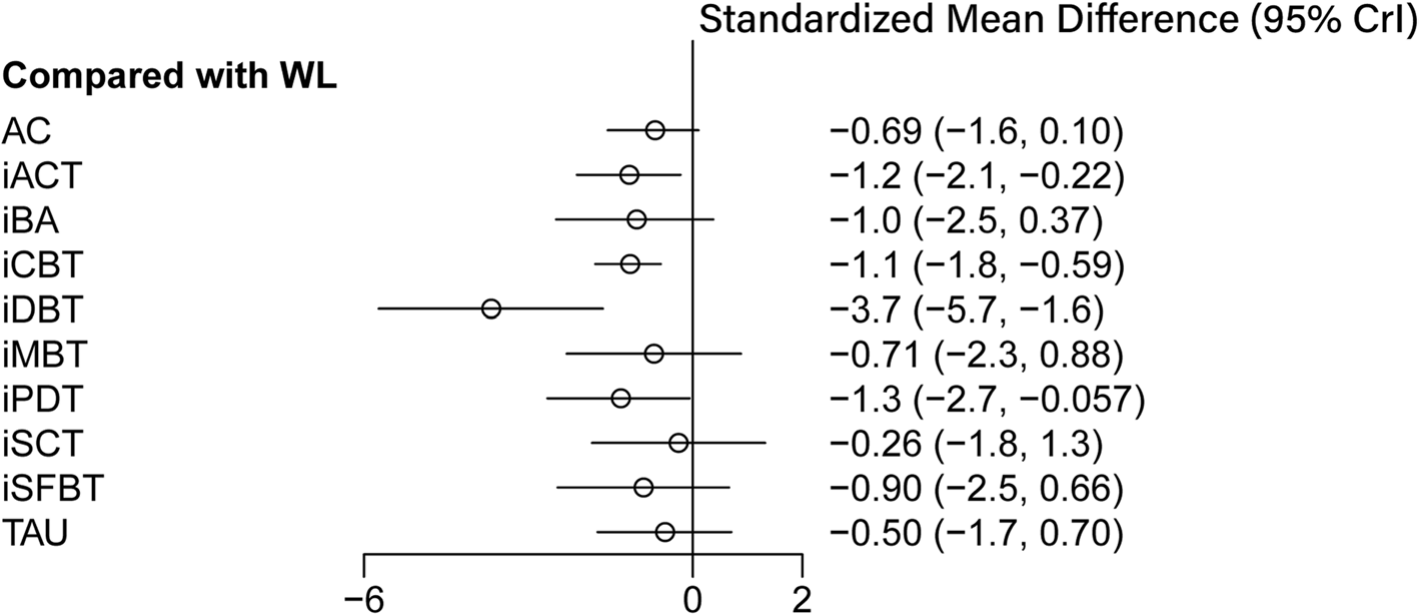
Forest plot of effectiveness

**Fig. 4:**
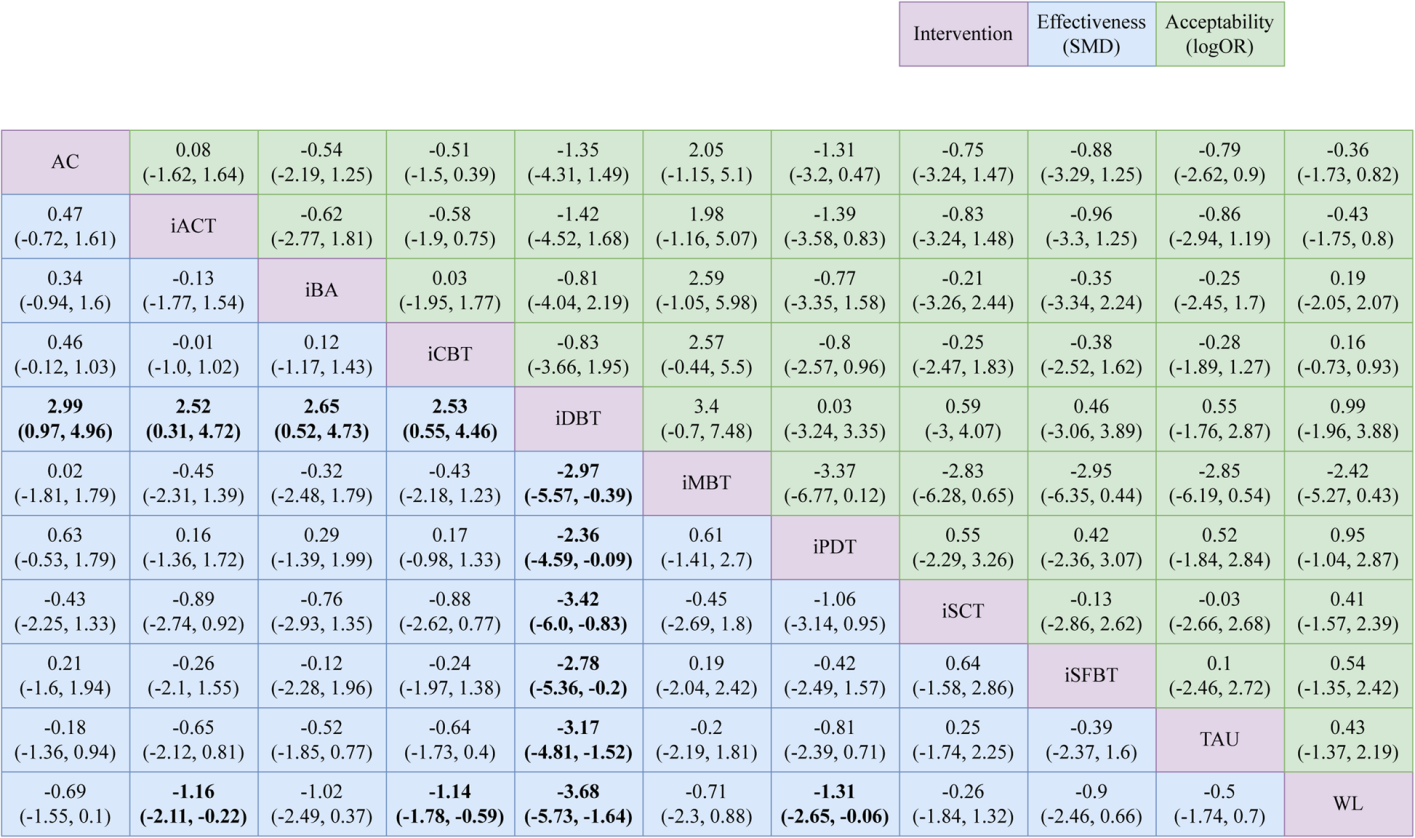
League table of effectiveness and acceptability. NOTE: AC = active control; iACT = internet-based acceptance and commitment therapy; iBA = internet-based behavioral activation; iCBT = internet-based cognitive behavioral therapy; iDBT = internet-based dialectical behavior therapy; iMBT = internet-based mindfulness-based therapy; iPDT = internet-based psychodynamic therapy; iSCT = internet-based social cognitive theory; iSFBT = internet-based solution-focused brief therapy; TAU = treatment as usual; WL = waiting list

As per the forest plot depicted in Fig. 3, iACT, iCBT, iDBT, and iPDT demonstrated statistically significant superiority over the WL control group (iACT [SMD = −1.2, CrI: −2.1 to −0.22], iCBT [SMD = −1.1, CrI: −1.8 to −0.59], iDBT [SMD = −3.7, CrI: −5.7 to −1.6], iPDT [SMD = −1.3, CrI: −2.7 to −0.057]). Notably, iDBT emerged as the most effective intervention among all the options considered. Compared to TAU, the majority of interventions exhibited effect sizes ranging from small to large (SMD ranging from −0.2 to −0.81). Similarly, when compared to AC, the interventions generally exhibited small to moderate effect sizes (SMD ranging from −0.21 to −0.63). For a comprehensive overview, refer to Fig. 4 and the SUCRA effectiveness chart provided in Additional File 7. The ranking of interventions based on SUCRA values is as follows: iDBT, iPDT, iCBT, iACT, iBA, iSFBT, iMBT, AC, TAU, iSCT, and WL (Table 1).

**Table 1:** Surface under the cumulative ranking curve (SUCRA) value of interventions

Intervention	SUCRA of effectiveness	SUCRA of acceptability
AC	0.3839	0.7063
iACT	0.6305	0.7097
iBA	0.5622	0.4747
iCBT	0.6502	0.4682
iDBT	0.9902	0.2744
iMBT	0.4257	0.9412
iPDT	0.6885	0.2318
iSCT	0.2564	0.4020
iSFBT	0.5007	0.3550
TAU	0.3144	0.3863
WL	0.0974	0.5504

*AC* active control, *iACT* internet-based acceptance and commitment therapy, *iBA* internet-based behavioral activation, *iCBT* internet-based cognitive behavioral therapy, *iDBT* internet-based dialectical behavior therapy, *iMBT* internet-based mindfulness-based therapy, *iPDT* internet-based psychodynamic therapy, *iSCT* internet-based social cognitive theory, *iSFBT* internet-based solution-focused brief therapy, *TAU* treatment as usual, *WL* waiting list

### NMA of acceptability

**Fig. 5:**
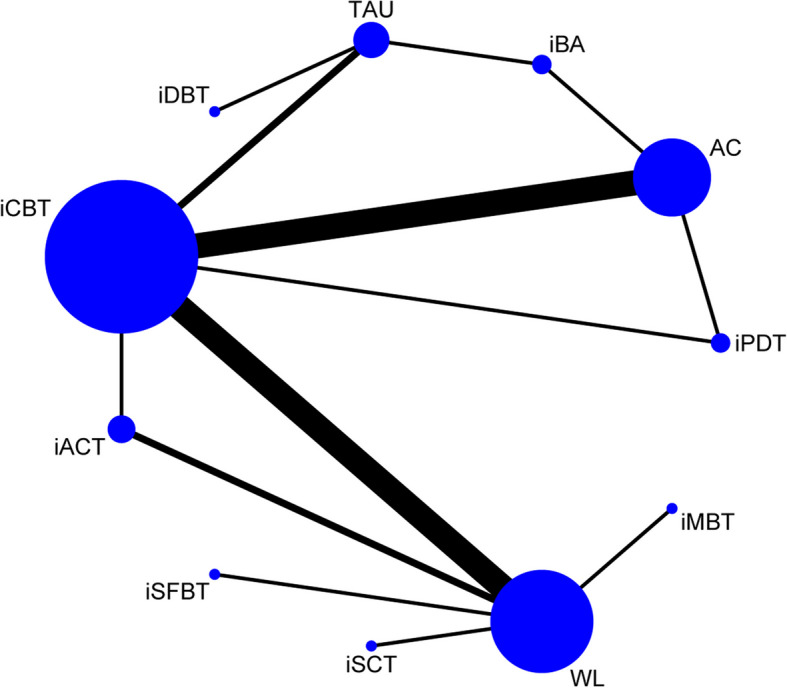
Network plot of acceptability

The network plot of acceptability is displayed in Fig. 5, which mirrors the structure of the effectiveness network plot, as all studies reported dropout rates. The Brooks-Gelman-Rubin diagnosis plot (Additional File 8) shows that the consistency model achieved good convergence. No significant global inconsistency was detected, as the DIC was similar to the DIC in the UME model (45.83 vs. 48.11). The node-splitting method also did not identify any significant local inconsistencies (Additional File 9).

**Fig. 6:**
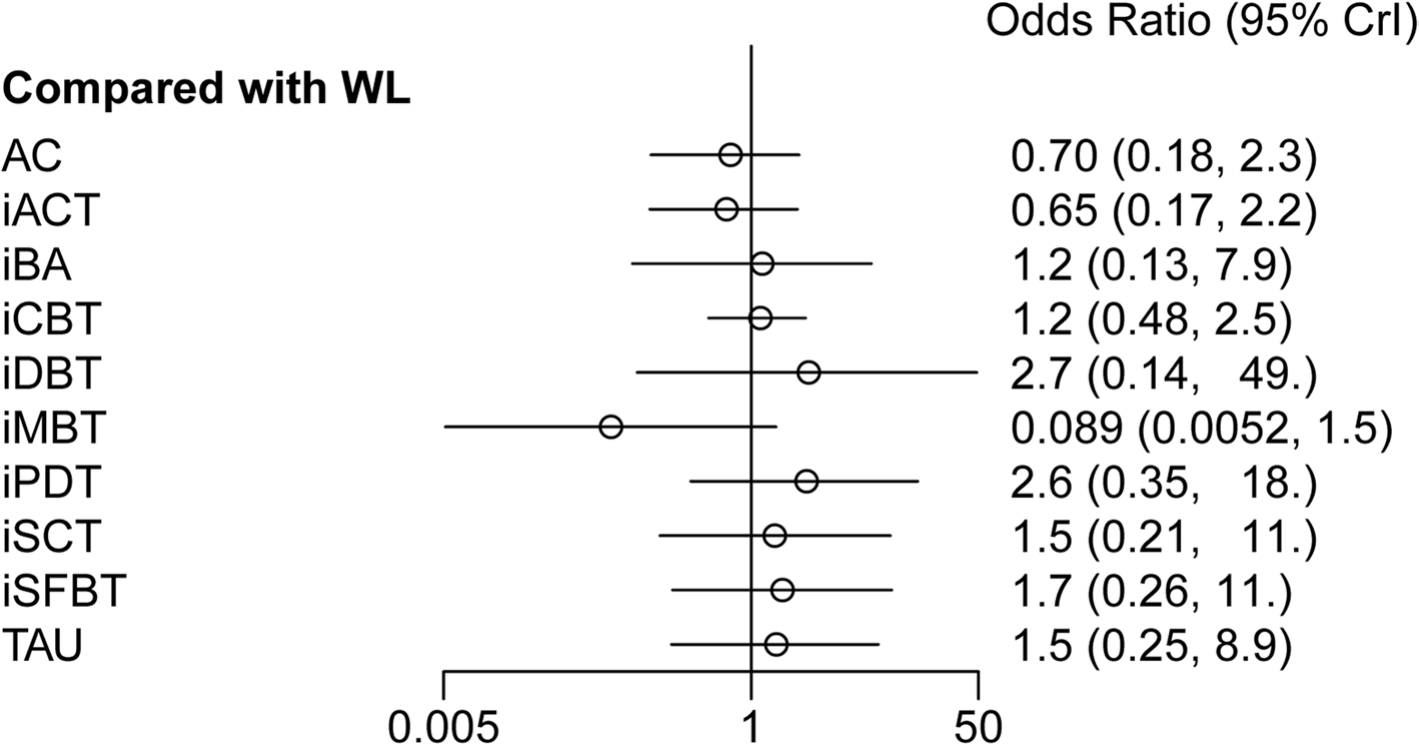
Forest plot of acceptability

**Fig. 7:**
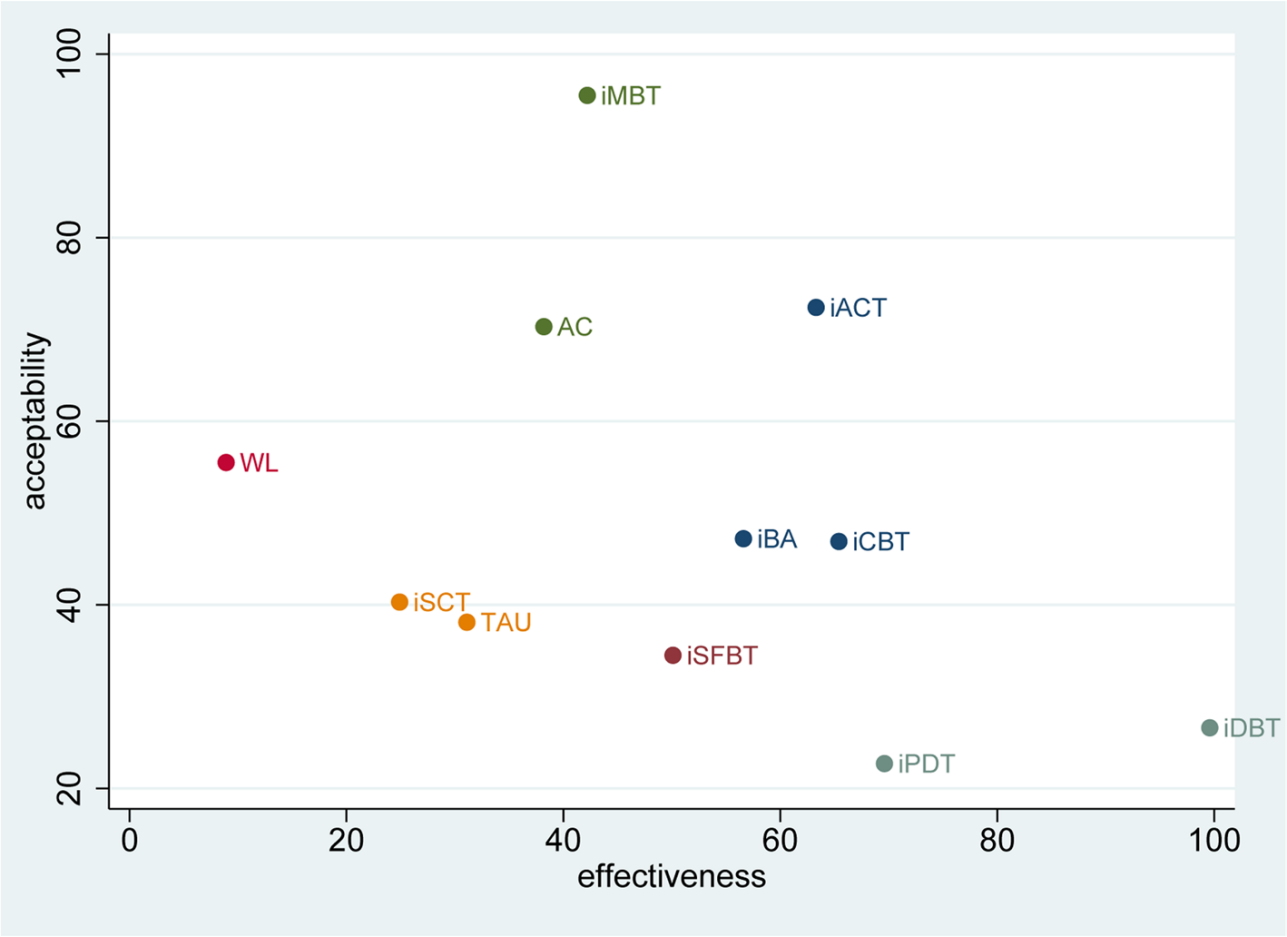
Coordinate of effectiveness and acceptability

According to the forest plot (Fig. 6), none of the interventions were significantly more acceptable than the WL control group. Similarly, no interventions were found to be significantly superior to others in terms of acceptability (Fig. 4). For a detailed visualization, refer to the SUCRA acceptability chart provided in Additional File 10. The ranking of interventions based on SUCRA values is as follows: iMBT, iACT, AC, WL, iBA, iCBT, iSCT, TAU, iSFBT, iDBT, and iPDT. Besides, we generated a coordinate plot where the x-axis represents the SUCRA values of effectiveness and the y-axis represents the SUCRA values of acceptability (Fig. 7). This plot intuitively displays the relative performance of each intervention. Interventions located closer to the upper right corner of the plot indicate better performance in both effectiveness and acceptability.

## Discussion

In recent decades, the prevalence of depression has been on the rise [60]. A global meta-analysis revealed that 27.6% of the population reported experienced depression during the COVID-19 pandemic [61]. Regionally, the prevalence in Asia (35.3%) was slightly higher than in Europe (32.4%) [62]. Given the limited access to psychological treatment resources, internet-based psychological treatments have become an important supplement and alternative to face-to-face interventions [63]. iCBT, with over 30 years of development, is one of the most popular forms of internet-based treatment and has been shown to be as effective as traditional face-to-face CBT [64]. This is likely why most of the studies we included focused on iCBT. Due to our strict inclusion criteria, the number of studies on other eligible internet-based interventions was quite limited. Our analysis was strictly limited to studies involving only patients with depression, while other studies included patients with both depression and/or anxiety disorders. Participants diagnosed solely with anxiety and not depression were excluded because our analysis focused exclusively on depression. However, it is important to acknowledge that depression is common in individuals with other mental disorders or physical diseases [65].

According to the results of a recent meta-analysis [13], we originally expected no significant difference between iCB and both WL and TAU. However, our analysis revealed a significant effect size for iCBT compared to WL, but it did not reach significance compared to AC, which contradicted Wu’s findings [13]. This discrepancy can be attributed to the inclusion of additional types of internet-based psychological interventions beyond iCBT in our research. Although the effectiveness of iCBT may not be as significant in young people as in adults, its importance as a flexible, time- and place-independent psychotherapy cannot be ignored. By customizing iCBT according to the characteristics of young people, it may yield better results.

In our NMA, iDBT was the only intervention that demonstrated statistical significance compared to TAU and AC. However, other interventions exhibited small to moderate effect sizes when compared to AC (SMD ranging from −0.21 to −0.63, excluding iMBT [0.02] and iSCT [0.43]), and small to large effect sizes compared to TAU (SMD ranging from −0.2 to −0.81, except iSCT [0.25]). Given the limited number of studies in our analysis, we speculate that with more studies, these interventions may achieve statistical significance. As depicted in Fig. 4, the effect sizes of iMBT versus AC, TAU, and WL were −0.02, −0.2, and −0.71, respectively. A meta-analysis by Reangsing et al. [66] assessed iMBT versus control groups (TAU and WL) in university students and found that iMBT significantly reduced depressive symptoms (SMD = 0.184, CrI: 0.024 to 0.344). Although the number of studies was limited, this somewhat supported our findings. In line with another systematic review evaluating the effectiveness of SFBT [67], SFBT was found to be equally effective as short-term PDT in adult, but for adolescents, SFBT only showed a positive trend in reducing depressive symptoms. Our iSFBT research results aligned with these findings. Regarding iSCT, we found no other studies specifically examining its effectiveness for adolescent depression. Research on SCT-based interventions for depression in young people is scarce. One study showed that college students who received SCT preventive group counseling interventions had reduced positive symptoms of emotional and/or anxiety disorders [68]. However, in the study we analyzed, there was no significant difference in mean depression scores between the iSCT group and the WL group after intervention (*p* = 0.062) [47]. We believe that the format and implementation of iSCT may require further exploration and optimization.

Dropout is a significant concern in psychological treatment, especially in internet-based interventions. Melville et al. reported that the weighted average dropout rate for internet-based psychological treatment programs was as high as 31% [19]. A meta-review indicated that patients with depression had higher dropout rates compared to those with anxiety disorders [69]. We believe that dropout in internet-based interventions is not solely the responsibility of psychotherapists. These interventions are delivered via various mediums such as websites, applications, and video games, involving multiple domains including psychology, visual communication design, software engineering, and AI in chatbots. Therefore, policymakers should play a pivotal role in establishing guidelines to regulate internet-based interventions and collaborate with experts from various fields to develop more suitable treatment plans for delivery via the internet.

We firmly believe that by strictly defining inclusion criteria to focus on patients with depression and integrating both effectiveness and acceptability into a coordinate system for analysis, our NMA can provide more reasonable and comprehensive results. Therapists prioritizing treatment efficacy may lean towards selecting iDBT, while those emphasizing patient acceptability may favor iMBT. For those seeking an optimal balance between efficacy and acceptability, iACT emerges as an ideal option. Our research findings exhibit robust generalizability and hold substantial reference value for related studies across diverse regions, including Asia, Europe, North America, South America, and Oceania.

Our research also has several limitations. Firstly, with the exception of iCBT (*n* = 18), the number of research records for each intervention measure was relatively limited (iDBT, iSFBT, iMBT, iSCT: *n* = 1; iBA, iPDT: *n* = 2; iACT: *n* = 3). The inclusion of additional studies could potentially enhance the reliability and persuasiveness of our findings. Besides, given that data collection was restricted to the end of treatment, we are unable to provide insights into long-term follow-up effects. Finally, it is noteworthy that all participants in our study were Chinese scholars, and despite our efforts to minimize cultural differences and language barriers, there remains a possibility of misinterpretation of literature written in English.

In conclusion, internet-based psychological interventions still face many challenges. Future research should endeavor to conduct comprehensive assessments of the efficacy of different types and forms of internet-based psychological interventions. Moreover, interdisciplinary collaboration among experts in various fields is essential to enhance both the effectiveness of internet-based psychological interventions and the overall user experience, thereby offering a superior intervention experience to a broader patient population.

## Conclusions

According to our NMA results, iACT, iCBT, iDBT, and iPDT demonstrated significantly better efficacy compared to WL. However, aside from iDBT, none of the other interventions showed statistically significant improvements over AC and TAU. Meanwhile, there was no significant difference in acceptability among all interventions. Based on SUCRA analysis, iDBT was identified as the most effective intervention with the highest probability of treating depression, while iMBT performed the best in terms of acceptability. However, considering the limitations of eligible studies, further investigations are needed to explore effectiveness and applicability of internet-based psychological interventions.

## Supplementary Information

Additional file 1. Search strategy.

Additional file 2. Study characteristics.

Additional file 3. Descriptions and details of interventions.

Additional file 4. Risk of bias.

Additional file 5. Brooks-Gelman-Rubin results of effectiveness.

Additional file 6. Nodesplit results of effectiveness.

Additional file 7. SUCRA of effectiveness.

Additional file 8. Brooks-Gelman-Rubin results of acceptability.

Additional file 9. Nodesplit results of acceptability.

Additional file 10. SUCRA of acceptability.

## Data Availability

No datasets were generated or analysed during the current study.

## Abbreviations

CBT: Cognitive behavioral therapy
BA: Behavioral activation
MBT: Mindfulness-based therapy
ACT: Acceptance and commitment therapy
WL: Waiting list
AC: Active control
TAU: Treatment as usual
iCBT: Internet-based cognitive behavioral therapy
iBA: Internet-based behavioral therapy
iDBT: Internet-based dialectical behavior therapy
iPDT: Internet-based psychodynamic therapy
iMBT: Internet-based mindfulness-based therapy
iACT: Internet-based acceptance and commitment therapy
iSFBT: Internet-based solution-focused brief therapy
iSCT: Internet-based social cognitive theory
CNKI: China National Knowledge Infrastructure
RCTs: Randomized controlled trials
PICOS: Population, intervention, comparator, outcome, study design
AI: Artificial intelligence
NMA: Network meta-analysis
SMD: Standardized mean difference
CrI: Credible interval
SD: Standard deviation
SE: Standard error
OR: Odds ratio
ITT: Intention-to-treat analysis
MCM: Markov chain Monte Carlo
DIC: Deviance information criterion
UME: Unrelated mean effect
SUCRA: Surface Under the Cumulative Ranking curve
OVK: Op Volle Kracht
SPARX: Smart, Positive, Active, Realistic, X-factor thoughts
