# Supplementary material for: Comparative effectiveness and acceptability of internet-based psychological interventions on depression in young people: a systematic review and network meta-analysis
Source: BMC Psychiatry. 2025 Apr 2;25:321. doi: 10.1186/s12888-025-06757-9 (PMC11967053; doi:10.1186/s12888-025-06757-9)
Supplement: Supplementary file 1 — Additional file 1. Search strategy. [file 12888_2025_6757_MOESM1_ESM.docx]

Additional File 1. Table of search strategy

| Database |  |
| --- | --- |
| Web of Science | #1: TS=(depress*) OR TS=(major depressive disorder)  #2: TS=(psychotherapy) OR TS=(psychotherapeutic*) OR TS=(psychological intervention) OR TS=(psychotherapeutic intervention) OR TS=(psychological treatment) OR TS=(mental health intervention)  #3: TS=(CBT) OR TS=(cognitive behavioral therapy) OR TS=(CT) OR TS=(cognitive therapy) OR TS=(MBCT) OR TS=(mindfulness-based cognitive therapy) OR TS=(MBT) OR TS=(mindfulness-based therapy) OR TS=(MT) OR TS=(mindfulness therapy) OR TS=(BAT) OR TS=(BT) OR TS=(behavioral activation therapy) OR TS=(behavior therapy) OR TS=(ACT) OR TS=(acceptance and commitment therapy) OR TS=(IPT) OR TS=(interpersonal psychotherapy) OR TS=(PST) OR TS=(problem solving therapy) OR TS=(supportive psychotherapy) OR TS=(psychodynamic)  #4: #2 OR #3  #5: TS=(internet) OR TS=(internet-based) OR TS=(internet-delivered) OR TS=(web) OR TS=(web-based) OR TS=(mobile) OR TS=(mobile-based) OR TS=(computer) OR TS=(computer-assisted) OR TS=(digital) OR TS=(online) OR TS=(technology-based) OR TS=(emental) OR TS=(e-mental) OR TS=(ehealth) OR TS=(e-health) OR TS=(e-therapy) OR TS=(m-health)  #6: TS=(RCT) OR TS=(random*) OR TS=(randomized controlled trial)  #7: TS=(child*) OR TS=(adolescen*) OR TS=(youth*) OR TS=(young*) OR TS=(young adult) OR TS=(young person) OR TS=(young people) OR TS=(college*) OR TS=(student*) OR TS=(teen*) OR TS=(school) OR TS=(boy*) OR TS=(girl*) OR TS=(juvenile*)  #8: #1 AND #4 AND #5 AND #6 AND #7 and Chinese or English (Languages) Timespan: 1995-01-01 to 2024-07-31 |
| PubMed | #1: (depression[MeSH Terms]) OR (depressive disorder[MeSH Terms]) OR (depress*[Title/Abstract])  #2: (psychotherapy[MeSH Terms]) OR (psychotherapy[Title/Abstract]) OR (psychotherapeutic*[Title/Abstract]) OR (psychological intervention[Title/Abstract]) OR (psychotherapeutic intervention[Title/Abstract]) OR (psychological treatment[Title/Abstract]) OR (mental health intervention[Title/Abstract])  #3: (cognitive behavioral therapy[MeSH Terms]) OR (behavior therapy[MeSH Terms]) OR (acceptance and commitment therapy[MeSH Terms]) OR (mindfulness[MeSH Terms]) OR (interpersonal psychotherapy[MeSH Terms]) OR (psychotherapy, psychodynamic[MeSH Terms]) OR (CBT[Title/Abstract]) OR (CT[Title/Abstract]) OR (cognitive therapy[Title/Abstract]) OR (MBCT[Title/Abstract]) OR (mindfulness-based cognitive therapy[Title/Abstract]) OR (MBT[Title/Abstract]) OR (mindfulness-based therapy[Title/Abstract]) OR (MT[Title/Abstract]) OR (mindfulness therapy[Title/Abstract]) OR (BAT[Title/Abstract]) OR (behavioral activation therapy[Title/Abstract]) OR (ACT[Title/Abstract]) OR (IPT[Title/Abstract]) OR (PST[Title/Abstract]) OR (problem solving therapy[Title/Abstract]) OR (supportive psychotherapy[Title/Abstract])  #4: #2 OR #3  #5: (internet[Title/Abstract]) OR (internet-based[Title/Abstract]) OR (internet-delivered[Title/Abstract]) OR (web[Title/Abstract]) OR (web-based[Title/Abstract]) OR (mobile[Title/Abstract]) OR (mobile-based[Title/Abstract]) OR (computer[Title/Abstract]) OR (computer-assisted[Title/Abstract]) OR (digital[Title/Abstract]) OR (technology-based[Title/Abstract]) OR (online[Title/Abstract]) OR (emental[Title/Abstract]) OR (e-mental[Title/Abstract]) OR (ehealth[Title/Abstract]) OR (e-health[Title/Abstract]) OR (e-therapy[Title/Abstract]) OR (m-health[Title/Abstract])  #6: (random*[Title/Abstract]) OR (RCT[Title/Abstract]) OR (randomized controlled trial[Title/Abstract])  #7: (child*[Title/Abstract]) OR (adolescen*[Title/Abstract]) OR (youth*[Title/Abstract]) OR (young*[Title/Abstract]) OR (young adult*[Title/Abstract]) OR (young person[Title/Abstract]) OR (young people[Title/Abstract]) OR (college*[Title/Abstract]) OR (student*[Title/Abstract]) OR (teen*[Title/Abstract]) OR (school[Title/Abstract]) OR (boy[Title/Abstract]) OR (girl*[Title/Abstract]) OR (juvenile[Title/Abstract])  #8: #1 AND #4 AND #5 AND #6 AND #7  Filters: Chinese, English, from 1995/1/1 - 2024/12/1 |
| ScienceDirect | Title, abstract, keywords:  (depress)AND((internet)OR(digital)OR(online))AND((psychotherapy)OR(psychotherapeutic))AND(random)AND((adolescent)OR(student))  Year:1995-2024 |
| ProQuest | S1: mainsubject.Exact("depressive disorder" OR "depression") OR summary(depress*)  S2: mainsubject.Exact("psychotherapy") OR summary((psychotherapy) OR (psychotherapeutic*) OR (psychological intervention) OR (psychotherapeutic intervention) OR (psychological treatment) OR (mental health intervention))  S3: mainsubject.Exact("cognitive behavioral therapy" OR "behavior therapy" OR "mindfulness" OR "interpersonal psychotherapy" OR "acceptance and commitment therapy" OR "psychodynamic psychotherapy") OR summary((CBT) OR (CT) OR (cognitive therapy) OR (MBCT) OR (mindfulness-based cognitive therapy) OR (MBT) OR (mindfulness-based therapy) OR (MT) OR (mindfulness therapy) OR (BAT) OR (behavioral activation therapy) OR (ACT) OR (IPT) OR (PST) OR (problem solving therapy) OR (supportive psychotherapy))  S4: [S2] OR [S3]  S5: summary((internet) OR (internet-based) OR (internet-delivered) OR (web) OR (web-based) OR (mobile) OR (mobile-based) OR (computer) OR (computer-assisted) OR (digital) OR (technology-based) OR (online) OR (emental) OR (e-mental) OR (ehealth) OR (e-health) OR (e-therapy) OR (m-health))  S6: summary((random*) OR (RCT) OR (randomized controlled trial))  S7: summary((child*) OR (adolescen*) OR (youth*) OR (young*) OR (young adult*) OR (young person) OR (young people) OR (college*) OR (student*) OR (teen*) OR (school) OR (boy*) OR (girl*) OR (juvenile*))  S8: [S1] AND [S4] AND [S5] AND [S6] AND [S7] AND la.exact(“English” OR “Chinese”) AND pd(19950101-20241201) |
| Cochrane Library | #1: MeSH descriptor: [Depression] explode all trees  #2: MeSH descriptor: [Depressive Disorder] explode all trees  #3: (depress*):ti,ab,kw  #4: #1 OR #2 OR #3  #5: MeSH descriptor: [Psychotherapy] explode all trees  #6: (psychotherapy):ti,ab,kw OR (psychotherapeutic*):ti,ab,kw OR (psychological intervention):ti,ab,kw OR (psychotherapeutic intervention):ti,ab,kw OR (psychological treatment):ti,ab,kw OR (mental health intervention):ti,ab,kw  #7: #5 OR #6  #8: MeSH descriptor: [Cognitive Behavioral Therapy] explode all trees  #9: MeSH descriptor: [Behavior Therapy] explode all trees  #10: MeSH descriptor: [Acceptance and Commitment Therapy] explode all trees  #11: MeSH descriptor: [Mindfulness] explode all trees  #12: MeSH descriptor: [Interpersonal Psychotherapy] explode all trees  #13: MeSH descriptor: [Psychotherapy, Psychodynamic] explode all trees  #14: (CBT):ti,ab,kw OR (CT):ti,ab,kw OR (cognitive therapy):ti,ab,kw OR (MBCT):ti,ab,kw OR (mindfulness-based cognitive therapy):ti,ab,kw OR (MBT):ti,ab,kw OR (mindfulness-based therapy):ti,ab,kw OR (MT):ti,ab,kw OR (mindfulness therapy):ti,ab,kw OR (BAT):ti,ab,kw OR (behavioral activation therapy):ti,ab,kw OR (ACT):ti,ab,kw OR (IPT):ti,ab,kw OR (PST):ti,ab,kw OR (problem solving therapy):ti,ab,kw OR (supportive psychotherapy)  #15: #8 OR #9 OR #10 OR #11 OR #12 OR #13 OR #14  #16: #7 OR #15  #17: (internet):ti,ab,kw OR (internet-based):ti,ab,kw OR (internet-delivered):ti,ab,kw OR (web):ti,ab,kw OR (web-based):ti,ab,kw OR (mobile):ti,ab,kw OR (mobile-based):ti,ab,kw OR (computer):ti,ab,kw OR (computer-assisted):ti,ab,kw OR (digital):ti,ab,kw OR (technology-based):ti,ab,kw OR (online):ti,ab,kw OR (emental):ti,ab,kw OR (e-mental):ti,ab,kw OR (ehealth):ti,ab,kw OR (e-health):ti,ab,kw OR (e-therapy):ti,ab,kw OR (m-health)  #18: (random*):ti,ab,kw OR (RCT):ti,ab,kw OR (randomized controlled trial)  #19: (child*):ti,ab,kw OR (adolescen*):ti,ab,kw OR (youth*):ti,ab,kw OR (young*):ti,ab,kw OR (young adult*):ti,ab,kw OR (young person):ti,ab,kw OR (young people):ti,ab,kw OR (college*):ti,ab,kw OR (student*):ti,ab,kw OR (teen*):ti,ab,kw OR (school):ti,ab,kw OR (boy*):ti,ab,kw OR (girl*):ti,ab,kw OR (juvenile)  #20: #4 AND #16 AND #17 AND #18 AND #19  Range from 01/01/1995 to 01/12/2024 |
| CNKI | (SU%'抑郁')  AND  (SU%'网络' OR SU%'线上' OR SU%'互联网' OR SU%'远程' OR SU%'电子' OR SU%'电脑' OR SU%'手机' OR SU%'app' OR SU%'多媒体' OR SU%'数字')  AND  (SU%'心理' OR SU%'心理健康' OR SU%'心理咨询' OR SU%'心理治疗' OR SU%'心理干预' OR SU%'心理教育' OR SU%'认知行为' OR SU%'正念' OR SU%'行为激活' OR SU%'认知疗法' OR SU%'接纳与承诺' OR SU%'人际关系疗法' OR SU%'问题解决疗法' OR SU%'支持疗法')  AND  (SU%'随机对照试验' OR SU%'rct' OR SU%'随机')  AND  (SU%'青少年' OR SU%'儿童' OR SU%'青年' OR SU%'青春期' OR SU%'发育期' OR SU%'年轻人' OR SU%'学生')  AND  发表时间:1995-01-01到2024-12-01 |
| Wanfang | 中英文扩展&主题词扩展:  (主题:(抑郁)  and  主题:("网络" or "线上" or "互联网" or "远程" or "电子" or "电脑" or "手机" or "app" or "多媒体" or "数字")  and  主题:("心理" or "心理健康" or "心理咨询" or "心理治疗" or "心理干预" or "心理教育" or "认知行为" or "正念" or "行为激活" or "认知疗法" or "接纳与承诺" or "人际关系疗法" or "问题解决疗法" or "支持疗法")  and  主题:("随机对照试验" or "rct" or "随机")  and  主题:("青少年" or "儿童" or "青年" or "青春期" or "发育期" or "年轻人" or "学生"))  and 发表时间:1995-2024 |
| Embase | #1: 'depression'/exp OR 'depressive disorder'/exp OR depress*:ab,ti  #2: 'psychotherapy'/exp OR psychotherapy:ab,ti OR psychotherapeutic*:ab,ti OR 'psychological intervention':ab,ti OR 'psychotherapeutic intervention':ab,ti OR 'psychological treatment':ab,ti OR 'mental health intervention':ab,ti  #3: 'cognitive behavioral therapy'/exp OR 'behavior therapy'/exp OR 'acceptance and commitment therapy'/exp OR 'mindfulness'/exp OR 'interpersonal psychotherapy'/exp OR 'psychodynamic psychotherapy'/exp OR CBT:ab,ti OR CT:ab,ti OR 'cognitive therapy':ab,ti OR MBCT:ab,ti OR 'mindfulness-based cognitive therapy':ab,ti OR MBT:ab,ti OR 'mindfulness-bsaed therapy':ab,ti OR MT:ab,ti OR 'mindfulness therapy':ab,ti OR BAT:ab,ti OR 'behavioral activation therapy':ab,ti OR ACT:ab,ti OR IPT:ab,ti OR PST:ab,ti OR 'problem solving therapy':ab,ti OR 'supportive psychotherapy':ab,ti  #4: #2 OR #3  #5: internet:ab,ti OR internet-based:ab,ti OR internet-deliviered:ab,ti OR web:ab,ti OR web-based:ab,ti OR mobile:ab,ti OR mobile-based:ab,ti OR computer:ab,ti OR computer-assisted:ab,ti OR digital:ab,ti OR technology-based:ab,ti OR online:ab,ti OR emental:ab,ti OR e-mental:ab,ti OR ehealth:ab,ti OR e-health:ab,ti OR e-therapy:ab,ti OR m-health:ab,ti  #6: random*:ab,ti OR RCT:ab,ti OR 'randomized controlled trial':ab,ti  #7: child*:ab,ti OR adolescen*:ab,ti OR youth*:ab,ti OR young*:ab,ti OR 'young adult*':ab,ti OR 'young person':ab,ti OR 'young people':ab,ti OR college*:ab,ti OR student*:ab,ti OR teen*:ab,ti OR school:ab,ti OR boy*:ab,ti OR girl*:ab,ti OR juvenile:ab,ti  #8: #1 AND #4 AND #5 AND #6 AND #7 AND ([chinese]/lim OR [english]/lim) AND [1995-2024]/py |
