# Supplementary material for: Comparative effectiveness and acceptability of internet-based psychological interventions on depression in young people: a systematic review and network meta-analysis
Source: BMC Psychiatry. 2025 Apr 2;25:321. doi: 10.1186/s12888-025-06757-9 (PMC11967053; doi:10.1186/s12888-025-06757-9)
Supplement: Supplementary file 2 — Additional file 2. Study characteristics. [file 12888_2025_6757_MOESM2_ESM.docx]

Additional File 2. Table of study characteristics

| Study | Country | Measurement | Female  (%) | Intervention duration | Intervention | Randomized N | Baseline | | Post-treatment | | | Dropout |
| --- | --- | --- | --- | --- | --- | --- | --- | --- | --- | --- | --- | --- |
|  |  |  |  |  |  |  | Mean | SD | N | Mean | SD |  |
| Ren et al, 2016[1] | China | CES-D | 62.22 | 3 | iCBT | 47 | 27.67 | 1.43 | 47 | 21.66 | 1.58 | 13 |
|  |  |  |  |  | WL | 15 | 25.47 | 1.95 | 15 | 30.08 | 2.24 | 4 |
| Zhang et al, 2024[2] | China | MADRS | 59.82 | 48 | iDBT | 60 | 19.54 | 4.22 | 55 | 7.61 | 2.81 | 5 |
|  |  |  |  |  | TAU | 60 | 19.8 | 4.34 | 57 | 19.91 | 4.64 | 3 |
| Lindqvist et al, 2020[3] | Sweden | QIDS-A17-SR | 80 | 8 | iPDT | 38 | 15.45 | 3.25 | 33 | 9.3 | 5.08 | 5 |
|  |  |  |  |  | AC | 38 | 15.71 | 3.13 | 37 | 13.24 | 5.1 | 1 |
| Conley et al, 2024[4] | US | PHQ-9 | 89.7 | 8 | iMBT | 104 | 12.51 | 4.45 | 89 | 8.42 | 5.56 | 1 |
|  |  |  |  |  | WL | 41 | 11.65 | 4.64 | 31 | 12.23 | 4.69 | 4 |
| Topooco et al, 2018[5] | Sweden | BDI-II | 94.29 | 8 | iCBT | 33 | 33.1 | 9.4 | 33 | 19.9 | 7.2 | 3 |
|  |  |  |  |  | AC | 37 | 32.3 | 10.2 | 37 | 25.2 | 7.8 | 1 |
| Davis et al, 2023[6] | US | DASS-21 Depression | 77.17 | 10 | iCBT | 47 | 10.9 | 5.5 | 20 | 5.8 | 4.5 | 27 |
|  |  |  |  |  | iACT | 45 | 12.4 | 5.5 | 20 | 5.9 | 5.6 | 25 |
| Smith et al, 2015[7] | UK | MFQ-Child | NA | 8 | iCBT | 55 | 25.6 | 11.1 | 55 | 13.4 | 12.9 | 0 |
|  |  |  |  |  | WL | 57 | 24.8 | 11.8 | 55 | 24.3 | 13.6 | 2 |
| Sanabria, et al, 2020[8] | Colombia | PHQ-9 | 71 | 7 | iCBT | 107 | 14.22 | 2.81 | 22 | 8.33 | 5.71 | 86 |
|  |  |  |  |  | WL | 107 | 13.82 | 2.93 | 54 | 13.09 | 5.06 | 53 |
| Do et al, 2021[9] | Korea | PHQ-9 | NA | 5 | iCBT | 28 | 9.24 | 4.62 | 25 | 5.84 | 3.74 | 3 |
|  |  |  |  |  | WL | 27 | 11.92 | 6.87 | 25 | 10.44 | 6.28 | 2 |
|  |  |  |  |  |  |  |  |  |  |  |  |  |
| Study | Country | Measurement | Female  (%) | Intervention duration | Intervention | Randomized N | Baseline | | Post-treatment | | | Dropout |
|  |  |  |  |  |  |  | Mean | SD | N | Mean | SD |  |
| Kramer et al, 2014[10] | Netherlands | CES-D | 78.81 | 18 | iSFBT | 131 | 39.49 | 8.58 | 131 | 24.86 | 8.51 | 73 |
|  |  |  |  |  | WL | 132 | 39.74 | 7.13 | 132 | 33.09 | 9.69 | 56 |
| Zanden et al, 2012[11] | Netherlands | CES-D | 84.4 | 12 | iCBT | 121 | 32.5 | 8.4 | 121 | 19.3 | 9.7 | 25 |
|  |  |  |  |  | WL | 123 | 32.2 | 8.2 | 123 | 27 | 8.6 | 25 |
| Merry et al, 2012[12] | New Zealand | CDRS-R | 65.78 | 8 | iCBT | 94 | 43.02 | 11.12 | 94 | 33.92 | 11.19 | 9 |
|  |  |  |  |  | TAU | 93 | 42.09 | 10.38 | 93 | 35.07 | 9.71 | 8 |
| Zhao et al, 2022(a)[13] | China | BDI-II | 52.75 | 6 | iACT | 95 | 26.39 | 8.65 | 92 | 13.61 | 9.48 | 3 |
|  |  |  |  |  | WL | 87 | 26.76 | 9.53 | 76 | 26.05 | 10.06 | 11 |
| Topooco et al, 2019[14] | Sweden | BDI-II | 95.71 | 8 | iCBT | 35 | 31.6 | 10 | 35 | 16 | 11.3 | 4 |
|  |  |  |  |  | AC | 35 | 28.8 | 7.9 | 35 | 24.8 | 10.4 | 0 |
| Moeini et al, 2019[15] | Iran | CES-D | 100 | 24 | iSCT | 64 | 24.6 | 11.7 | 64 | 19.5 | 10.9 | 19 |
|  |  |  |  |  | WL | 64 | 22.3 | 11.8 | 64 | 22.5 | 12.1 | 14 |
| Schniering et al, 2022[16] | Australia | SMFQ-Y | 65.93 | 8 | iCBT | 45 | 14.4 | 6.1 | 45 | 8.59 | 11.2 | 5 |
|  |  |  |  |  | WL | 46 | 14.57 | 6.08 | 46 | 14.51 | 10.44 | 5 |
| Bell et al, 2023[17] | Australia | PHQ-8 | 61.82 | 6 | iCBT | 29 | 15.34 | 3.93 | 29 | 12.11 | 4.63 | 1 |
|  |  |  |  |  | WL | 26 | 15.65 | 4.03 | 26 | 14 | 4.59 | 2 |
| Stasiak et al, 2014[18] | New Zealand | CDRS-R | 41.18 | 7 | iCBT | 17 | 48.29 | 6.49 | 17 | 30.41 | 7.38 | 1 |
|  |  |  |  |  | AC | 17 | 45.47 | 9.44 | 17 | 36.29 | 13.77 | 4 |
| Study | Country | Measurement | Female  (%) | Intervention duration | Intervention | Randomized N | Baseline | | Post-treatment | | | Dropout |
|  |  |  |  |  |  |  | Mean | SD | N | Mean | SD |  |
| Peake et al, 2024[19] | US | PHQ-8 | 63.13 | 5 | iBA | 80 | 14.36 | 4.78 | 74 | 9.51 | 5.91 | 28 |
|  |  |  |  |  | AC | 79 | 13.29 | 4.51 | 79 | 10.43 | 5.39 | 14 |
| Srivastava et al, 2020[20] | India | BDI-II | 23.81 | 12 | iCBT | 11 | 26.4 | 3.4 | 10 | 10.5 | 1.4 | 1 |
|  |  |  |  |  | TAU | 10 | 24.3 | 3.8 | 9 | 15.7 | 5.9 | 1 |
| Andersson et al, 2022[21] | Sweden | CDRS-R | 59.38 | 11 | iBA | 21 | 53.58 | 9.79 | 21 | 36.8 | 11.04 | 1 |
|  |  |  |  |  | TAU | 11 | 53.2 | 9 | 11 | 44.9 | 10 | 2 |
| Ip et al, 2016[22] | China | CDRS-R | 68.1 | 32 | iCBT | 130 | 20.66 | 9.32 | 130 | 19.77 | 8.35 | 7 |
|  |  |  |  |  | AC | 127 | 20.43 | 9.43 | 127 | 21.72 | 9.71 | 0 |
| Poppelaars et al, 2016[23] | Netherlands | RADS-2 | 100 | 8 | iCBT | 51 | 62.61 | 11.97 | 51 | 57.88 | 12.57 | 4 |
|  |  |  |  |  | AC | 51 | 61.9 | 11.97 | 51 | 57.74 | 12.56 | 1 |
| Fitzpatrick et al, 2017[24] | US | PHQ-9 | 83.93 | 2 | iCBT | 34 | 14.3 | 6.65 | 31 | 11.14 | 3.95 | 3 |
|  |  |  |  |  | AC | 36 | 13.25 | 5.17 | 25 | 13.67 | 4.05 | 11 |
| Zhao et al, 2022(b)[25] | China | BDI-II | 48.39 | 6 | iACT | 63 | 25.9 | 10.02 | 63 | 14.97 | 10.3 | 5 |
|  |  |  |  |  | WL | 61 | 27.33 | 9.71 | 61 | 25.78 | 9.79 | 5 |
| Deady et al, 2016[26] | Australia | PHQ-9 | 59.6 | 5 | iCBT | 60 | 16.58 | 4.62 | 60 | 10.64 | 9.21 | 30 |
|  |  |  |  |  | AC | 44 | 15.95 | 5.38 | 44 | 14.53 | 7.45 | 18 |
| Mechler et al, 2022[27] | Sweden | QIDS-A17-SR | 83.46 | 10 | iCBT | 136 | 15.51 | 3.35 | 132 | 9.08 | 5.3 | 4 |
|  |  |  |  |  | iPDT | 136 | 15.63 | 3.43 | 129 | 8.79 | 5.83 | 7 |

Reference

1. 任志洪, 李献云, 赵陵波, 余香莲, 李政汉, 赖丽足, 阮怡君, 江光荣: 抑郁症网络化自助干预的效果及作用机制——以汉化MoodGYM为例. 心理学报 2016, 48(7):818-832.

2. 张艳萍, 严芳, 王海岭, 郭正军, 赵晶媛, 赵玉洁: “互联网+”辩证行为疗法干预模式在青少年非自杀性自伤干预中的应用研究. 中国全科医学 2024, 27(15):1825-1832.

3. Lindqvist K, Mechler J, Carlbring P, Lilliengren P, Falkenstrom F, Andersson G, Johansson R, Edbrooke-Childs J, Dahl H-SJ, Bergsten KL *et al*: Affect-Focused Psychodynamic Internet-Based Therapy for Adolescent Depression: Randomized Controlled Trial. Journal of Medical Internet Research 2020, 22(3).

4. Conley CS, Gonzales CH, Huguenel BM, Rauch AA, Kahrilas IJ, Duffecy J, Silton RL: Benefits of a technology-delivered mindfulness intervention for psychological distress and positive wellbeing in depressed college students: Post-intervention and follow-up effects from an rct. Mindfulness 2024.

5. Topooco N, Berg M, Johansson S, Liljethörn L, Radvogin E, Vlaescu G, Nordgren LB, Zetterqvist M, Andersson G: Chat- and Internet-based cognitive–behavioural therapy in treatment of adolescent depression: Randomised controlled trial. BJPsych Open 2018, 4(4):199-207.

6. Davis CH, Twohig MP, Levin ME: Choosing ACT or CBT: A preliminary test of incorporating client preferences for depression treatment with college students. Journal of Affective Disorders 2023, 325:413-420.

7. Smith P, Scott R, Eshkevari E, Jatta F, Leigh E, Harris V, Robinson A, Abeles P, Proudfoot J, Verduyn C *et al*: Computerised CBT for depressed adolescents: Randomised controlled trial. Behaviour research and therapy 2015, 73:104-110.

8. Salamanca-Sanabria A, Richards D, Timulak L, Connell S, Perilla MM, Parra-Villa Y, Castro-Camacho L: A Culturally Adapted Cognitive Behavioral Internet-Delivered Intervention for Depressive Symptoms: Randomized Controlled Trial. Jmir Mental Health 2020, 7(1).

9. Do R, Lee S, Kim J-S, Cho M, Shin H, Jang M, Shin M-S: Effectiveness and dissemination of computer-based cognitive behavioral therapy for depressed adolescents: Effective and accessible to whom? Journal of Affective Disorders 2021, 282:885-893.

10. Kramer J, Conijn B, Oijevaar P, Riper H: Effectiveness of a Web-Based Solution-Focused Brief Chat Treatment for Depressed Adolescents and Young Adults: Randomized Controlled Trial. Journal of Medical Internet Research 2014, 16(5):40-50.

11. Rianne van der Z: Effectiveness of an Online Group Course for Depression in Adolescents and Young Adults: A Randomized Trial. Journal of Medical Internet Research 2012, 14(3).

12. Merry SN, Stasiak K, Shepherd M, Frampton C, Fleming T, Lucassen MFG: The effectiveness of SPARX, a computerised self help intervention for adolescents seeking help for depression: randomised controlled non-inferiority trial. BMJ (Clinical research ed) 2012, 344:e2598-e2598.

13. Zhao C, Wampold BE, Ren Z, Zhang L, Jiang G: The efficacy and optimal matching of an internet‐based acceptance and commitment therapy intervention for depressive symptoms among university students: A randomized controlled trial in China. Journal of Clinical Psychology 2022, 78(7):1354-1375.

14. Topooco N, Byléhn S, Ellen Dahlström N, Holmlund J, Lindegaard J, Johansson S, Åberg L, Lise Bergman N, Zetterqvist M, Andersson G: Evaluating the Efficacy of Internet-Delivered Cognitive Behavioral Therapy Blended With Synchronous Chat Sessions to Treat Adolescent Depression: Randomized Controlled Trial. Journal of Medical Internet Research 2019, 21(11).

15. Moeini B, Bashirian S, Soltanian AR, Ghaleiha A, Taheri M: Examining the Effectiveness of a Web-Based Intervention for Depressive Symptoms in Female Adolescents: Applying Social Cognitive Theory. Journal of research in health sciences 2019, 19(3):e00454-e00454.

16. Schniering CA, Einstein D, Kirkman JJL, Rapee RM: Online treatment of adolescents with comorbid anxiety and depression: A randomized controlled trial. Journal of Affective Disorders 2022, 311:88-94.

17. Bell I, Arnold C, Gilbertson T, Simon DA, Castagnini E, Chen N, Nicholas J, Shaunagh OS, Valentine L, Alvarez-Jimenez M: A Personalized, Transdiagnostic Smartphone Intervention (Mello) Targeting Repetitive Negative Thinking in Young People With Depression and Anxiety: Pilot Randomized Controlled Trial. Journal of Medical Internet Research 2023, 25(1).

18. Stasiak K, Hatcher S, Frampton C, Merry SN: A pilot double blind randomized placebo controlled trial of a prototype computer-based cognitive behavioural therapy program for adolescents with symptoms of depression. Behavioural and Cognitive Psychotherapy 2014, 42(4):385-401.

19. Peake E, Miller I, Flannery J, Chen L, Lake J, Padmanabhan A: Preliminary efficacy of a digital intervention for adolescent depression: Randomized controlled trial. Journal of Medical Internet Research 2024, 26:22.

20. Srivastava P, Mehta M, Sagar R, Ambekar A: Smartteen- a computer assisted cognitive behavior therapy for Indian adolescents with depression- a pilot study. Asian Journal of Psychiatry 2020, 50:10.

21. Andersson R, Ahlen J, Mataix-Cols D, Lenhard F, Henje E, Mansson C, Sahlin H, Beckman M, Serlachius E, Vigerland S: Therapist-guided and self-guided internet-delivered behavioural activation for adolescents with depression: a randomised feasibility trial. Bmj Open 2022, 12(12).

22. Ip P, Chim D, Chan KL, Li TMH, Ho FKW, Van Voorhees BW, Tiwari A, Tsang A, Chan CWL, Ho M *et al*: Effectiveness of a culturally attuned Internet-based depression prevention program for Chinese adolescents: A randomized controlled trial. Depression and Anxiety 2016, 33(12):1123-1131.

23. Poppelaars M, Tak YR, Lichtwarck-Aschoff A, Engels RCME, Lobel A, Merry SN, Lucassen MFG, Granic I: A randomized controlled trial comparing two cognitive-behavioral programs for adolescent girls with subclinical depression: A school-based program (Op Volle Kracht) and a computerized program (SPARX). Behaviour research and therapy 2016, 80:33-42.

24. Fitzpatrick KK, Darcy A, Vierhile M: Delivering Cognitive Behavior Therapy to Young Adults With Symptoms of Depression and Anxiety Using a Fully Automated Conversational Agent (Woebot): A Randomized Controlled Trial. JMIR Ment Health 2017, 4(2):e19.

25. Zhao C, Ren Z, Jiang G, Zhang L: Mechanisms of change in an Internet-Based ACT study for depression in China. Journal of Contextual Behavioral Science 2022, 24:51-59.

26. Deady M, Mills KL, Teesson M, Kay-Lambkin F: An Online Intervention for Co-Occurring Depression and Problematic Alcohol Use in Young People: Primary Outcomes From a Randomized Controlled Trial. Journal of Medical Internet Research 2016, 18(3).

27. Mechler J, Lindqvist K, Carlbring P, Topooco N, Falkenstrom F, Lilliengren P, Andersson G, Johansson R, Midgley N, Edbrooke-Childs J *et al*: Therapist-guided internet-based psychodynamic therapy versus cognitive behavioural therapy for adolescent depression in Sweden: a randomised, clinical, non-inferiority trial. Lancet Digital Health 2022, 4(8):E594-E603.
