# Supplementary material for: Comparative effectiveness and acceptability of internet-based psychological interventions on depression in young people: a systematic review and network meta-analysis
Source: BMC Psychiatry. 2025 Apr 2;25:321. doi: 10.1186/s12888-025-06757-9 (PMC11967053; doi:10.1186/s12888-025-06757-9)
Supplement: Supplementary file 3 — Additional file 3. Descriptions and details of interventions. [file 12888_2025_6757_MOESM3_ESM.docx]

Additional File 3. Definitions of included interventions

| Intervention | Description and content of intervention |
| --- | --- |
| Acceptance and commitment therapy (ACT) | **Description:**  Contextual behavioral science (CBS) is the foundation of ACT and emphasizes the “act in context”. Thus, ACT seeks to alter the context in which behaviors occur, rather than seeking to target specific cognitions and emotions to alter their downstream effects on these behaviors (e.g., restructuring self-critical thoughts to decrease depressed mood and increase social activation).[1]  **Internet-based intervention:**   1. E-Book: *The Mindfulness and Acceptance Workbook for Depression: Using Acceptance and Commitment Therapy to Move Through Depression and Create a Life Worth Living* 2. Unguided self-help program.   Use pictures, text, audio, videos, and animation to convey the treatment principles of ACT to participants. The intervention program consisted of six units, each lasting about 30 min: (1) Depression is not terrible, realizing the truth of depression; (2) Refusing avoidance, accepting something when you cannot change it; (3) Cognitive fusion, improving and controlling your life; (4) Self-awareness, becoming a psychological observer of yourself; (5) Living in the present moment, practicing mindfulness; and (6) Living according to your own values, committing to act. |
| Behavioral activation (BA) | **Description:**  BA is based on a functional analytic model of depression. Its focus centers on identifying behaviors that are important within the individual’s context, advance the individual’s goals and experience of mastery or pleasure, and counter avoidance.[2]  **Internet-based intervention:**   1. The Spark app. It includes a 5-level program focused on providing psychoeducational content and delivering the BA model of depression by teaching 2 core skills (mood activity logging and activity scheduling). 2. Through a secure online platform, the intervention provides treatment rationale and psychoeducation, activity monitoring, activity scheduling, contingency management, values and goal assessments, and skills training in problem-solving and communication skills, relaxation techniques and relapse prevention. Also targets verbal and avoidance behaviors. Each session consists of eight chapters with age-appropriate text, animations, films and various exercises. |
| Cognitive behavioral therapy (CBT) | **Description:**  CBT is a combination of cognitive therapy and behavior therapy. It aims at evaluating, challenging, and modifying a patient’s dysfunctional beliefs.[3]  **Internet-based intervention:**   1. Software: MoodGYM, SPARX, Mello, Woebot, Smartteen 2. E-Book: *The Cognitive Behavioral Workbook for Depression: A Step-by-Step Guide to Overcoming Depression* 3. Web-based or computer-based program: *Yo puedo sentirme bien* (I can feel better), Depression-Alcohol (DEAL) Project, Stressbusters, *Hangbok-Nuri* (Happiness World) program, *Grip op Je Dip* (Master Your Mood, MYM)   These software and programs incorporate animation, text, sound, videos, interactive exercises and mini games. Treatment components include: (1) Psychoeducation; (2) Behavioral activation; (3) Cognitive restructuring; (4) Challenging core beliefs, and (5) relapse prevention. |
| Dialectical behavior therapy (DBT) | **Description:**  DBT is defined by dialectics, treatment strategies, and treatment targets. It focuses on enhancing dialectical thinking patterns to replace rigid, dichotomous thinking. And the underlying problem to treat is pervasive emotion dysregulation, which leads to impulsive and maladaptive behaviors.[4]  **Internet-based intervention:**  Real time video lessons by trainers on an online platform.  The treatment contains four modules: (1) Mindfulness training with 6 lessons and homework; (2) Interpersonal effectiveness training with 6 lessons and homework; (3) Emotion regulation training with 8 lessons and homework; and (4) Pain tolerance training with 11 lessons and homework |
| Mindfulness-based therapy (MBT) | **Description:**  MBT aims at foster greater mindful awareness, which means using one’s attention to monitor one’s moment-to-moment experience through an open lens of equanimity and acceptance. It focuses on helping individuals learn how to mindfully attend to body sensations and emotional reactions through the use of guided exercises (e.g., stretching, meditation).[5]  **Internet-based intervention:**  An online/mobile app that delivers brief, guided mindfulness exercises: Headspace. The app includes meditation courses that typically include 10 to 30 sessions following a particular theme (e.g., Handling Sadness, Managing Anxiety). Also includes single mindfulness meditation sessions that are intended to be used in a specific situation (e.g., difficult conversations), as well as guided mindful activities (e.g., mindful eating, mindful walking). And participants were invited to attend a 90-min orientation session where researchers briefly described the principles and benefits of mindfulness. |
| Psychodynamic therapy (PDT) | **Description:**  PDT focuses on expression and experience of affect. It includes three main objectives: defense restructuring (recognizing and relinquishing maladaptive defenses), affect restructuring (desensitization of affects through exposure to conflicted feeling), and self/other restructuring (improvement in sense of self and relationship with others).[6] Participants were encouraged to become aware of their own defenses, notice and regulate anxiety, and gradually approach previously warded off feelings related to situations that could trigger depressive symptoms.  **Internet-based intervention:**  A guided self-help program with therapist support and weekly chat sessions  Intervention consisted of 8 therapist-supported self-help modules on a secure online platform. Modules consisted of texts, videos, and a series of experiential exercises. Participants are encouraged to reflect on and experience underlying emotional conflicts that give rise to and perpetuate depressive symptoms. The intervention also focuses on noticing when anxiety is too high, anxiety regulation, and avoidance of emotions (defences). The aim of treatment is to achieve greater insights into the underlying emotional avoidance. The final part of the program contains material on how to talk about and share emotions in close relationships. |
| Social cognitive theory (SCT) | **Description:**  SCT is a behavioral health theory that explains behaviors in terms of a triadic and reciprocal model, in which an individual’s behavior, personal factors and the environment interact and influence each other. The specific constructs of SCT include self-efficacy, outcome expectations, social support, and goal-setting.[7]  **Internet-based intervention:**  The DAD (Dorehye Amozeshie Dokhtaran) internet intervention program. It contains 7 core modules including introduction and assessment, awareness-raising, positive psychology, problem-solving, thoughts and feelings, relaxation, physical exercise and lifestyle modifications. Each module was planned in a multimedia format that includes videos, real-life examples, pictures, animations, and assignments. |
| Solution-focused brief therapy (SFBT) | **Description:**  SFBT is grounded in the constructivist approaches to communication and social interactional theories. The active ingredients in SFBT includes conversations that involve a therapeutic process of co-constructing, by altering and/or creating new meanings with clients. And the specific questioning techniques (e.g., miracle questions, scaling, etc.) are an important means of facilitating changes with clients.[8]  **Internet-based intervention:**  A web-based anonymous SFBT chat intervention. The chat consists of individual real-time chat sessions with a trained health care professional in a secured chat room. During the sessions, SFBT techniques are used by the therapist, starting with asking the “miracle questions”, setting goals, looking for strengths or solutions, keeping the focus on what is going well or better, giving compliments, looking for exceptions to the problem, and asking the client to indicate on scales from 1-10 what progress is made in obtaining goals. |
| Active control (AC) | **Description:**  AC group receive active intervention such as psycho-education and basic support.  **Internet-based intervention:**   1. Attention control app or program consists of psycho-education, supportive contact, and weekly assessment. 2. E-Book about depression. |
| Treatment as usual (TAU) | **Description:**  TAU group receive pharmacotherapy and active psychological therapy (psycho-education, supportive interventions, stress management). Face-to-face therapy is allowed in TAU group.  **Internet-based intervention:**  At youth clinics, school-based counselling services, or their local CAMHS (children and adolescent mental health services), participants receive whether psycho-educational, pharmacological or a combination of both. |
| Waiting list (WL) | **Description:**  WL group receive no treatment during a study.  **Internet-based intervention:**  This group do not receive treatment until the end of treatment of intervention group. |

**Reference**

1. Twohig MP, Levin ME: Acceptance and Commitment Therapy as a Treatment for Anxiety and Depression: A Review. Psychiatr Clin North Am 2017, 40(4):751-770.

2. McCauley E, Gudmundsen G, Schloredt K, Martell C, Rhew I, Hubley S, Dimidjian S: The Adolescent Behavioral Activation Program: Adapting Behavioral Activation as a Treatment for Depression in Adolescence. J Clin Child Adolesc Psychol 2016, 45(3):291-304.

3. Nathan Thoma, Brian Pilecki, McKay D: Contemporary Cognitive Behavior Therapy A Review of Theory, History, and Evidence. Psychodyn Psychiatry 2015, 43(3):423-461.

4. DeCou CR, Comtois KA, Landes SJ: Dialectical Behavior Therapy Is Effective for the Treatment of Suicidal Behavior A Meta-Analysis. Behav Ther 2019, 50(1):60-72.

5. Creswell JD, Lindsay EK, Villalba DK, Chin B: Mindfulness Training and Physical Health: Mechanisms and Outcomes. Psychosom Med 2019, 81(3):224-232.

6. Johansson R, Bjorklund M, Hornborg C, Karlsson S, Hesser H, Ljotsson B, Rousseau A, Frederick RJ, Andersson G: Affect-focused psychodynamic psychotherapy for depression and anxiety through the Internet: a randomized controlled trial. PeerJ 2013, 1:e102.

7. Moeini B, Bashirian S, Soltanian AR, Ghaleiha A, Taheri M: Examining the Effectiveness of a Web-Based Intervention for Depressive Symptoms in Female Adolescents: Applying Social Cognitive Theory. Journal of research in health sciences 2019, 19(3):e00454-e00454.

8. Franklin C, Zhang A, Froerer A, Johnson S: Solution Focused Brief Therapy: A Systematic Review and Meta-Summary of Process Research. J Marital Fam Ther 2017, 43(1):16-30.
