# Supplementary material for: Comparative effectiveness and acceptability of internet-based psychological interventions on depression in young people: a systematic review and network meta-analysis
Source: BMC Psychiatry. 2025 Apr 2;25:321. doi: 10.1186/s12888-025-06757-9 (PMC11967053; doi:10.1186/s12888-025-06757-9)
Supplement: Supplementary file 4 — Additional file 4. Risk of bias. [file 12888_2025_6757_MOESM4_ESM.pdf]

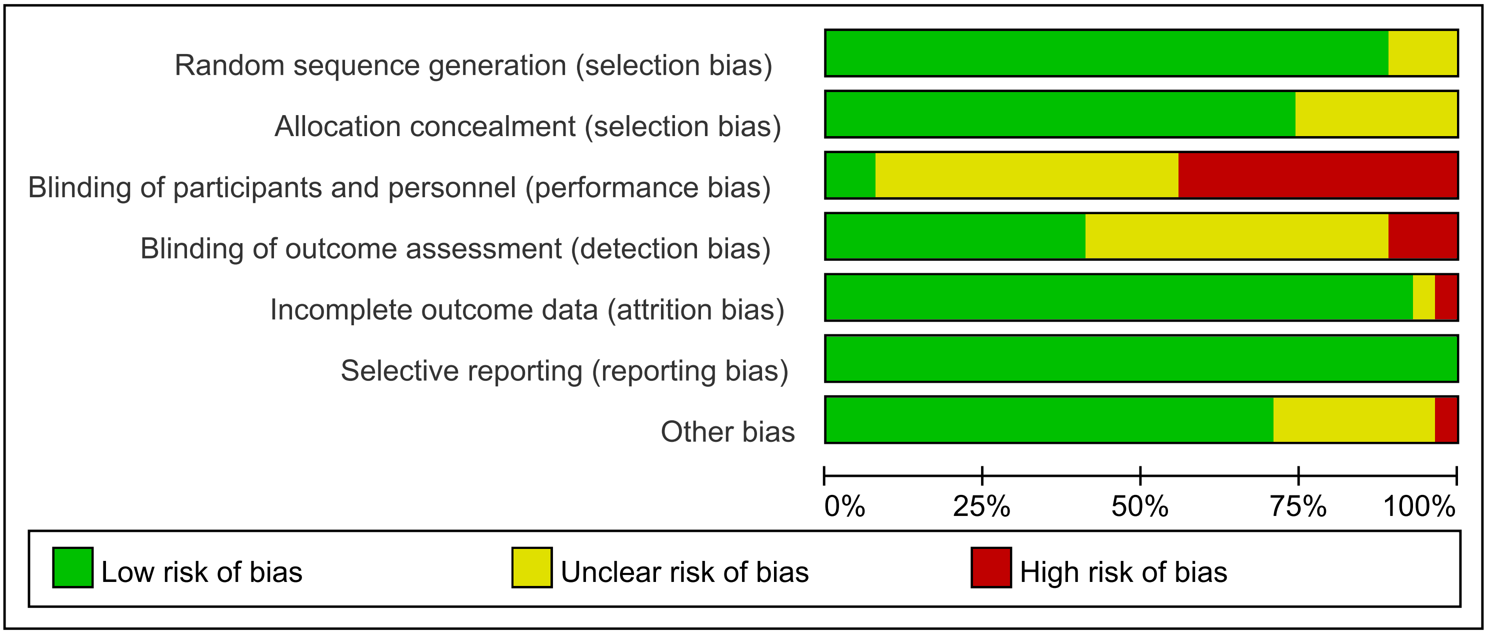
Random sequence generation (selection bias)
Allocation concealment (selection bias)
Blinding of participants and personnel (performance bias)
Blinding of outcome assessment (detection bias)
Incomplete outcome data (attrition bias)
Selective reporting (reporting bias)
Other bias
0%
25%
50%
75%
100%
Low risk of bias
Unclear risk of bias
High risk of bias

	Random sequence generation (selection bias)	Allocation concealment (selection bias)	Blinding of participants and personnel (performance bias)	Blinding of outcome assessment (detection bias)	Incomplete outcome data (attrition bias)	Selective reporting (reporting bias)	Other bias
Alicia Salamanca-Sanabria, 2020	+	+	?	+	+	+	+
Babak Moeini, 2019	?	?	?	?	+	+	+
Carolyn A. Schniering, 2022	+	+	?	?	−	+	+
Carter H. Davis, 2023	+	+	−	+	+	+	−
Chunxiao Zhao, 2022 (a)	?	?	?	?	+	+	+
Chunxiao Zhao, 2022 (b)	+	+	?	?	+	+	+
Colleen S. Conley, 2024	+	?	?	?	+	+	?
Emily Peake, 2024	?	?	?	+	+	+	+
Imogen Bell, 2023	+	+	−	−	+	+	+
Jakob Mechler, 2022	+	+	−	+	+	+	+
Jeannet Kramer, 2014	+	+	−	+	+	+	?
Karin Lindqvist, 2020	+	+	−	−	+	+	+
Karolina Stasiak, 2014	+	+	+	+	+	+	?
Kathleen Kara Fitzpatrick, 2017	+	+	−	?	+	+	?
Mark Deady, 2016	+	+	+	?	+	+	+
Marlou Poppelaars, 2016	+	+	−	?	+	+	+
Naira Topooco, 2018	+	+	−	?	+	+	+
Naira Topooco, 2019	+	+	?	−	+	+	+
Paakhi Srivastava, 2020	+	+	−	?	+	+	+
Patrick Ip, 2016	+	+	?	+	+	+	+
Patrick Smith, 2015	+	?	?	?	+	+	?
Rebecca Andersson, 2022	+	+	?	+	+	+	+
Rianne van der Zanden, 2012	+	+	?	+	+	+	?
Ryemi Do, 2021	+	+	−	+	+	+	+
Sally N Merry, 2012	+	+	−	+	+	+	+
Yanping Zhang, 2024	+	?	−	?	?	+	?
Zhihong Ren, 2016	+	?	?	?	+	+	+
