# Supplementary figures and images for: Comparative effectiveness and acceptability of internet-based psychological interventions on depression in young people: a systematic review and network meta-analysis
Source: BMC Psychiatry. 2025 Apr 2;25:321. doi: 10.1186/s12888-025-06757-9 (PMC11967053; doi:10.1186/s12888-025-06757-9)

**d.AC.iBA**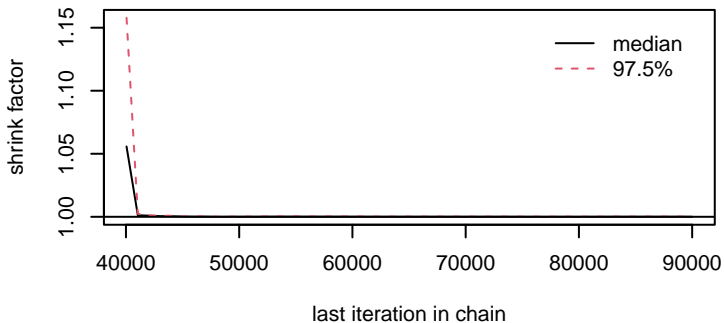**d.iCBT.AC**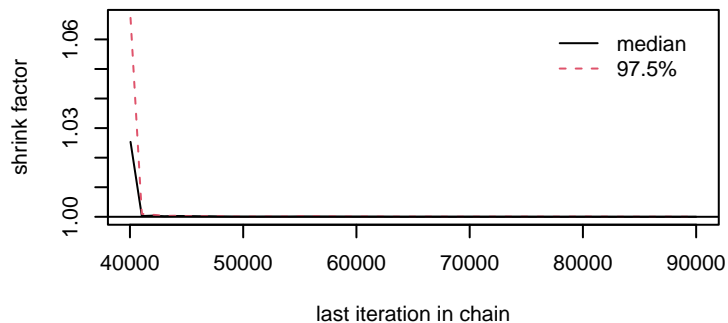**d.iCBT.iACT**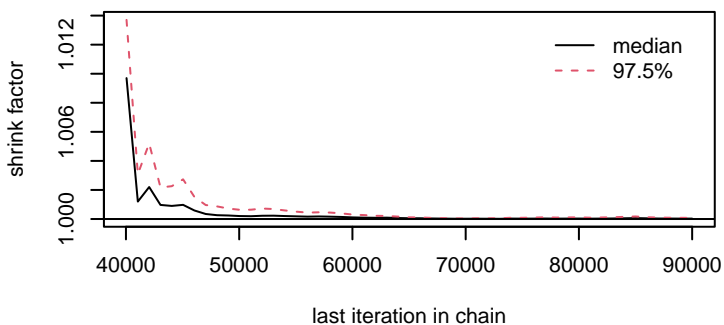**d.iCBT.iPDT**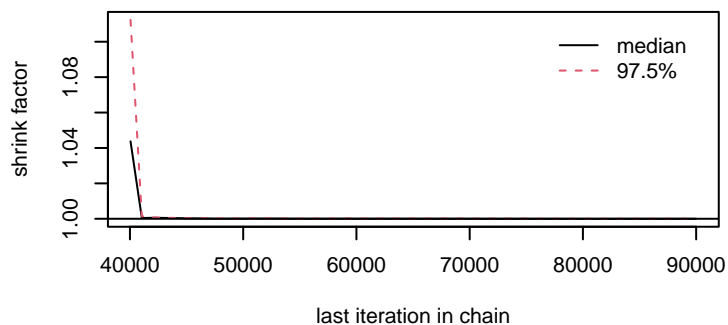**d.iCBT.TAU**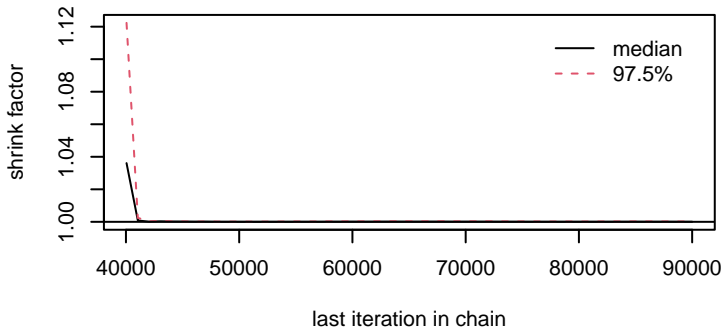**d.iCBT.WL**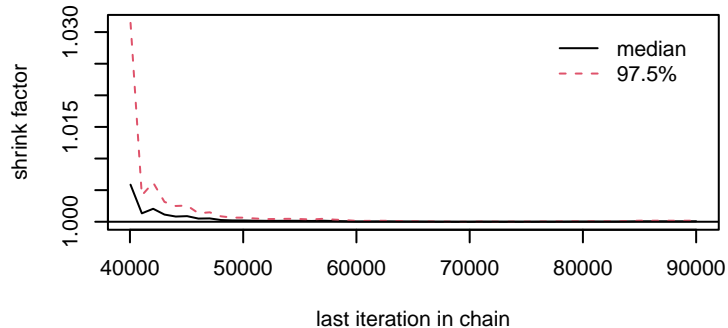

**d.TAU.iDBT**

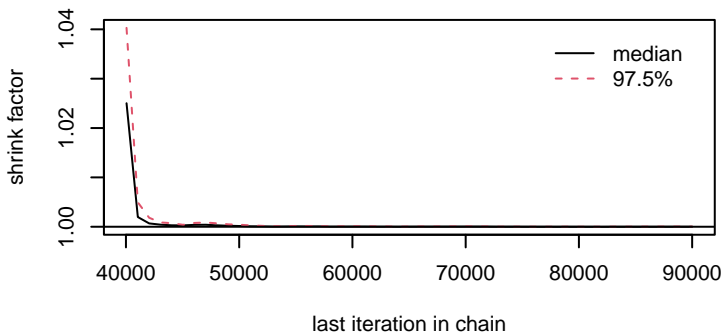

**d.WL.iMBT**

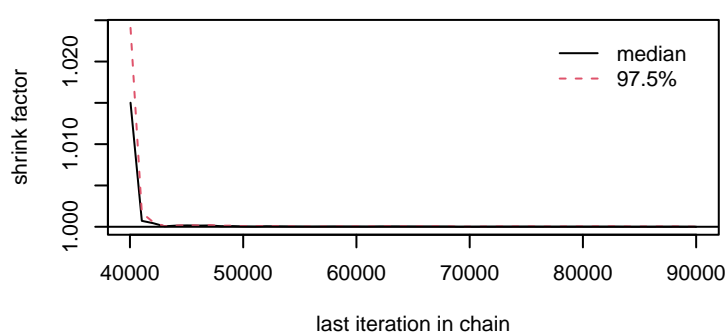

**d.WL.iSCT**

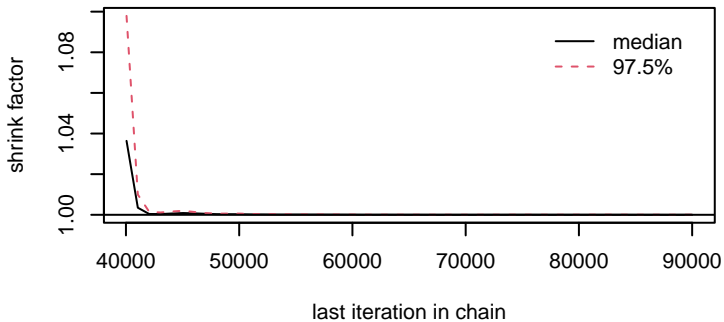

**d.WL.iSFBT**

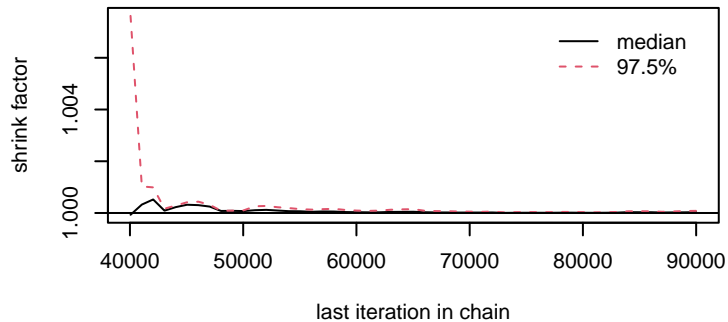

**sd.d**

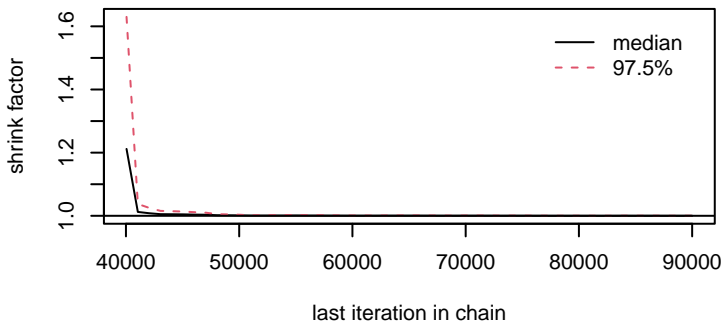

Supplement: Supplementary file 5 — Additional file 5. Brooks-Gelman-Rubin results of effectiveness. [file 12888_2025_6757_MOESM5_ESM.pdf]

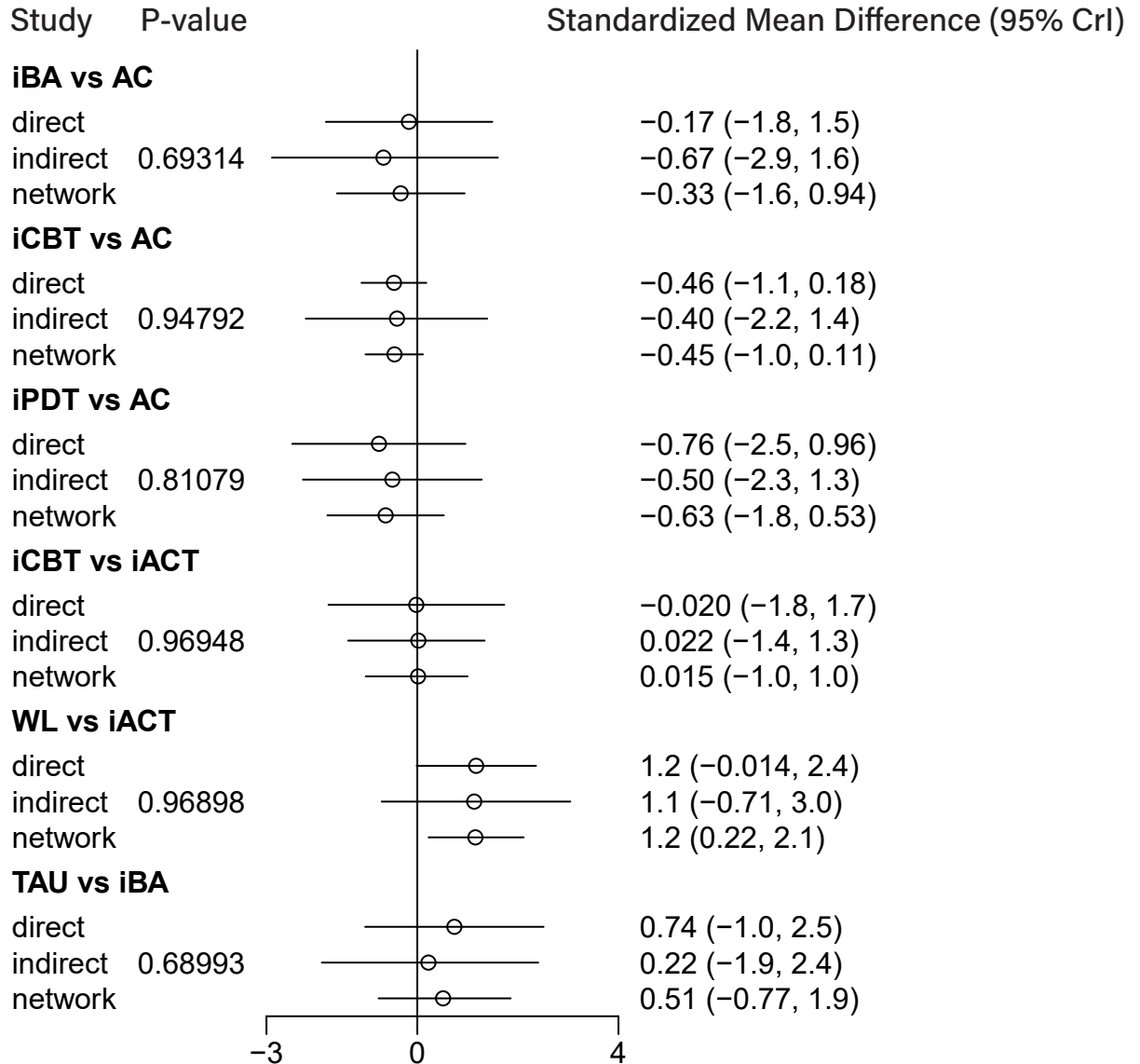

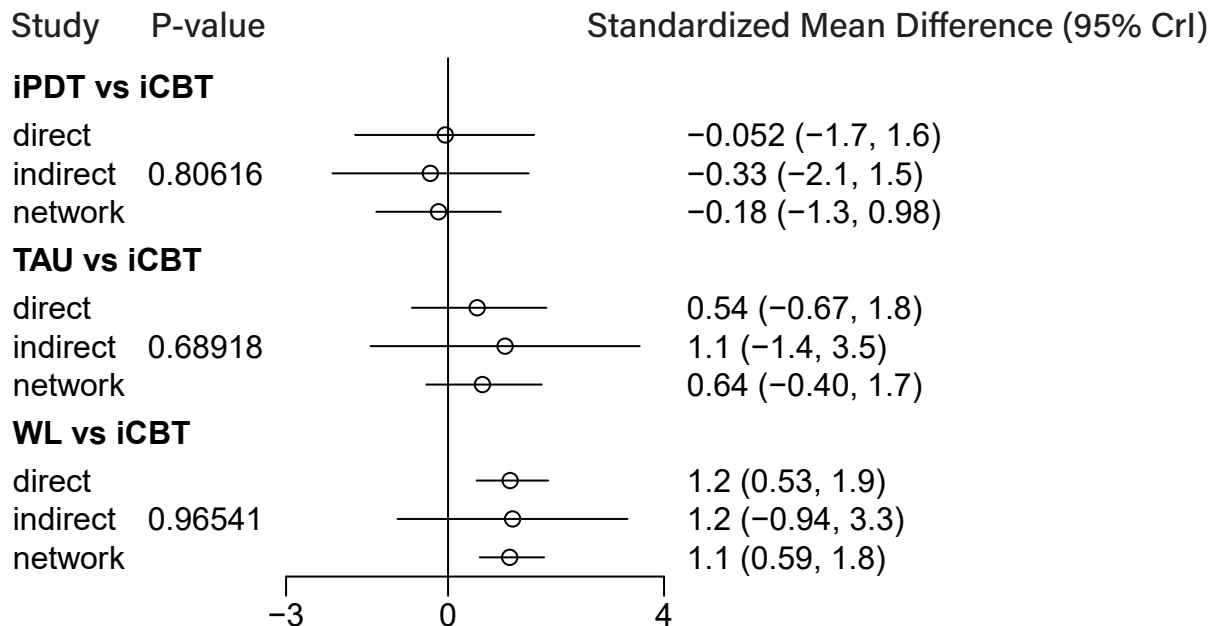

Supplement: Supplementary file 6 — Additional file 6. Nodesplit results of effectiveness. [file 12888_2025_6757_MOESM6_ESM.pdf]

Cumulative Probabilities

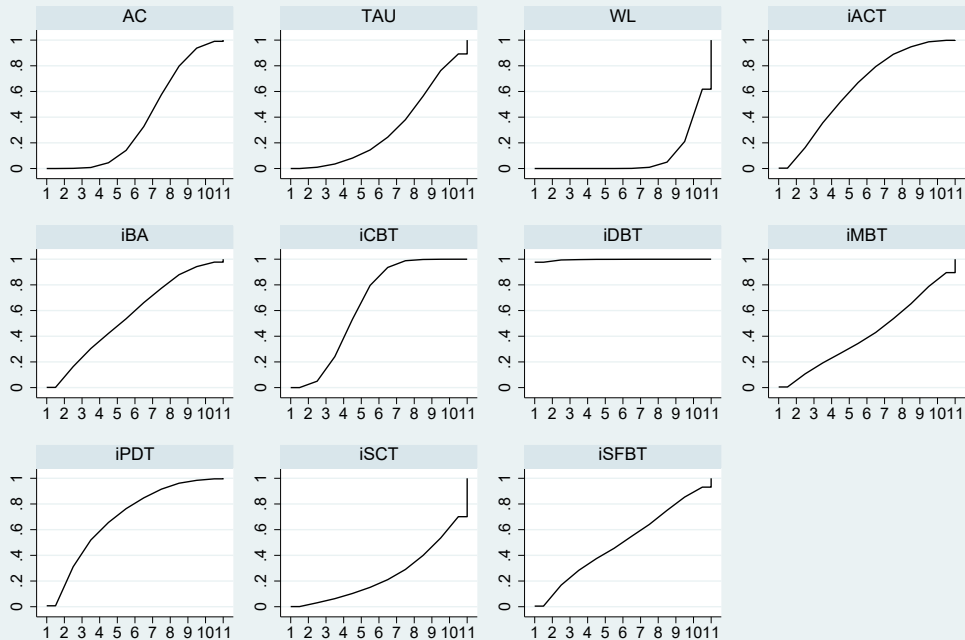

Rank

Graphs by Treatment

Supplement: Supplementary file 7 — Additional file 7. SUCRA of effectiveness. [file 12888_2025_6757_MOESM7_ESM.pdf]

**d.AC.iBA**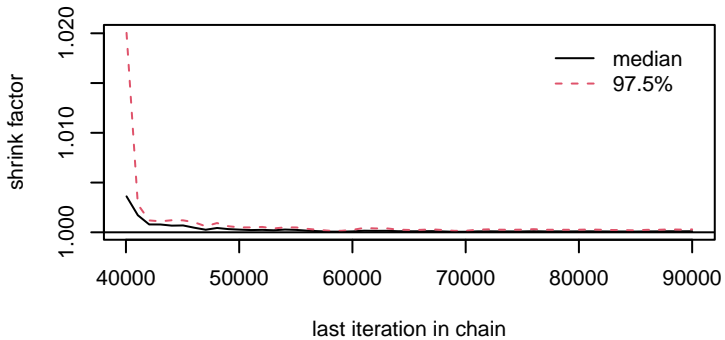**d.iCBT.AC**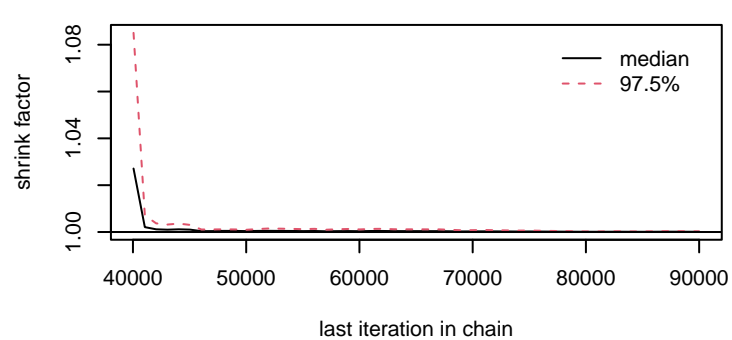**d.iCBT.iACT**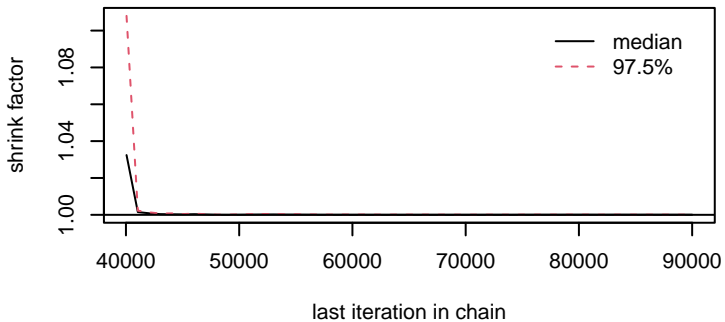**d.iCBT.iPDT**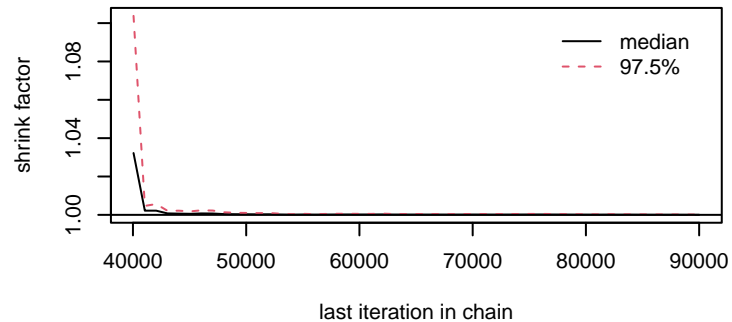**d.iCBT.TAU**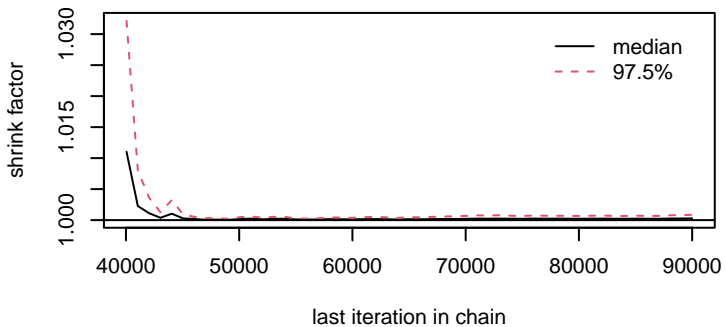**d.iCBT.WL**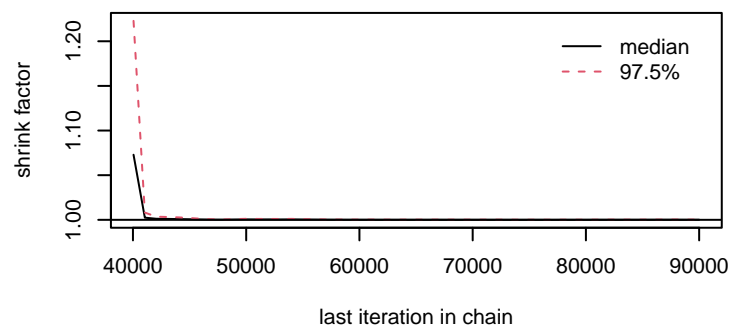

**d.TAU.iDBT**

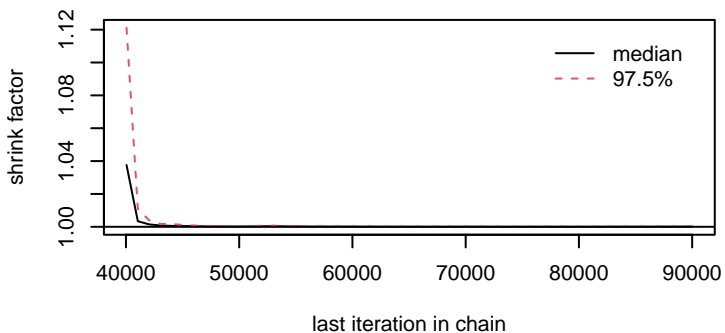

**d.WL.iMBT**

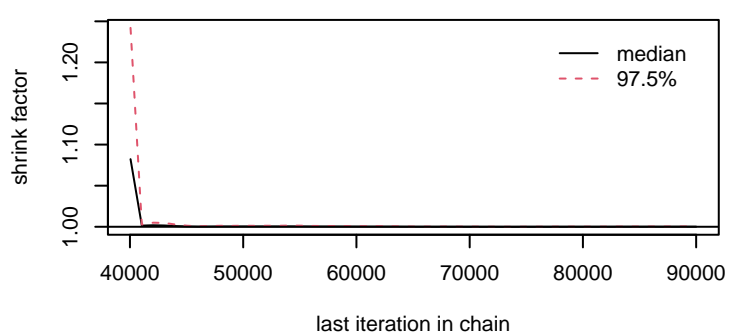

**d.WL.iSCT**

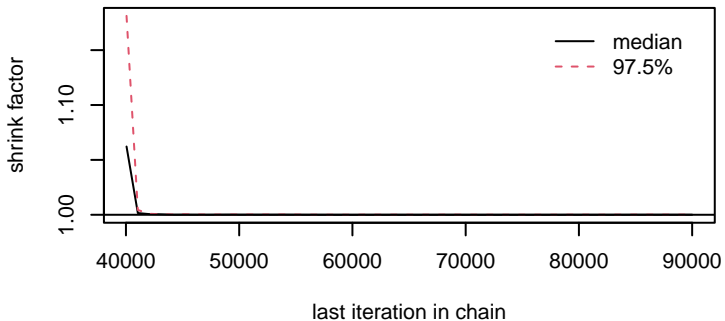

**d.WL.iSFBT**

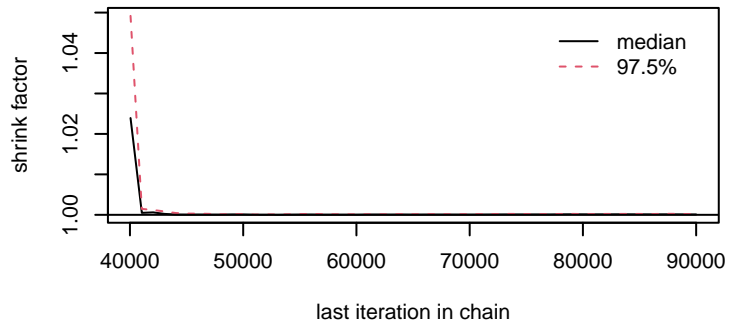

**sd.d**

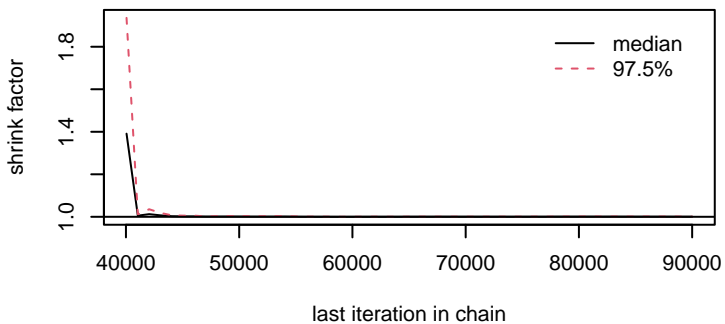

Supplement: Supplementary file 8 — Additional file 8. Brooks-Gelman-Rubin results of acceptability. [file 12888_2025_6757_MOESM8_ESM.pdf]

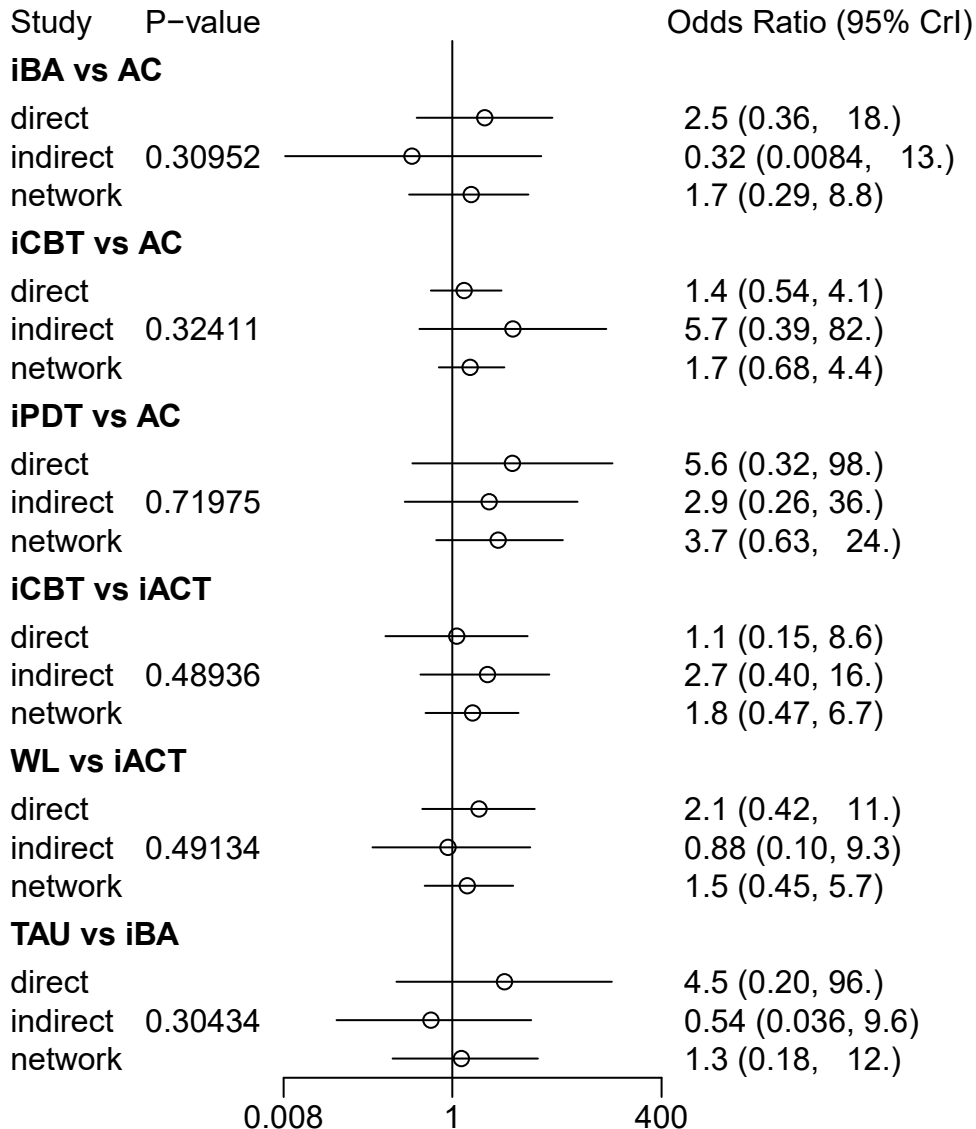

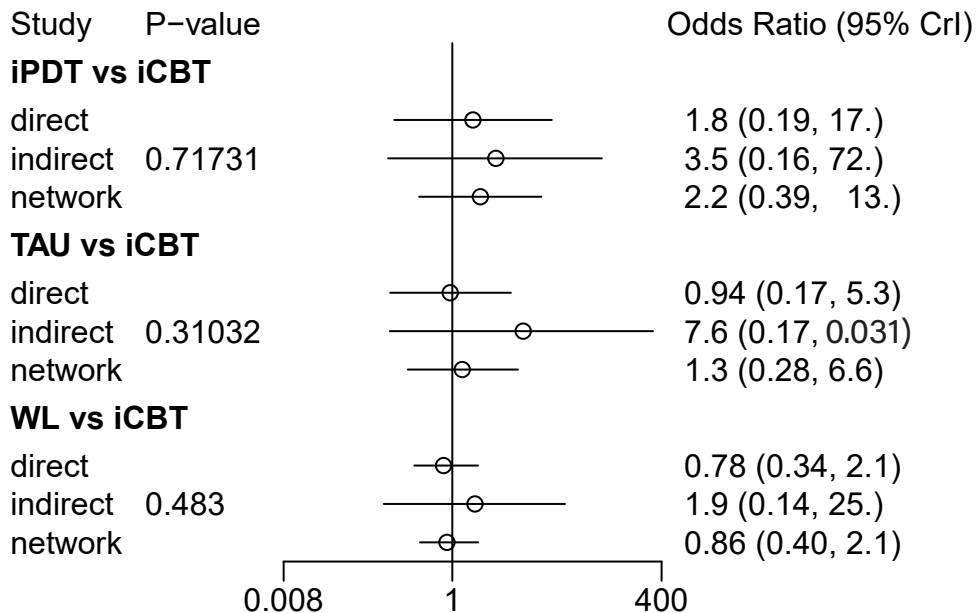

Supplement: Supplementary file 9 — Additional file 9. Nodesplit results of acceptability. [file 12888_2025_6757_MOESM9_ESM.pdf]

Cumulative Probabilities

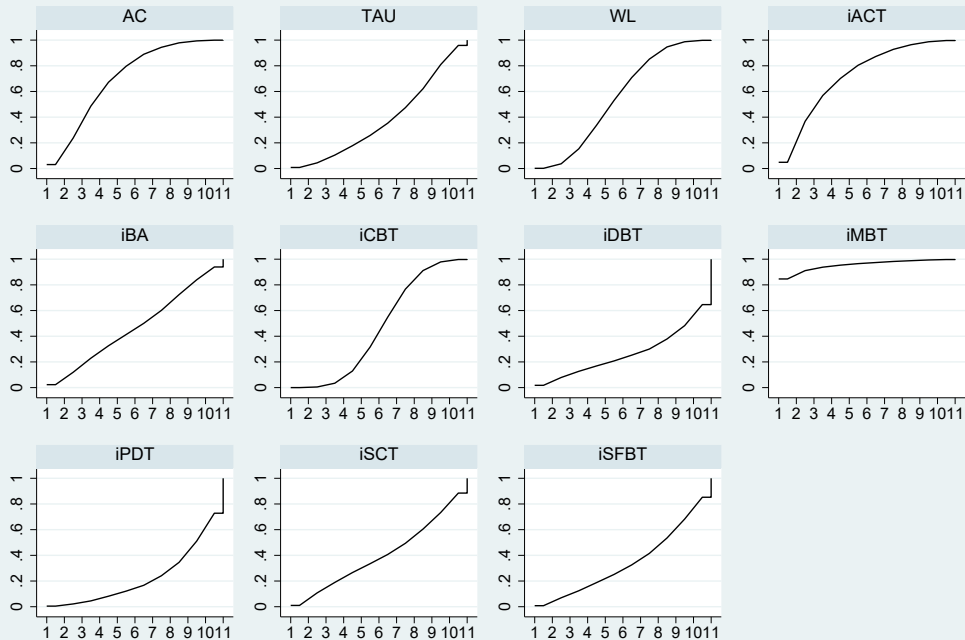

Rank

Graphs by Treatment

Supplement: Supplementary file 10 — Additional file 10. SUCRA of acceptability. [file 12888_2025_6757_MOESM10_ESM.pdf]
